# Histone deacetylase 1 and 2 drive differentiation and fusion of progenitor cells in human placental trophoblasts

**DOI:** 10.1038/s41419-020-2500-6

**Published:** 2020-05-04

**Authors:** Gargi Jaju Bhattad, Mariyan J. Jeyarajah, Megan G. McGill, Vanessa Dumeaux, Hiroaki Okae, Takahiro Arima, Patrick Lajoie, Nathalie G. Bérubé, Stephen J. Renaud

**Affiliations:** 1 Department of Anatomy and Cell Biology, Schulich School of Medicine and Dentistry, University of Western Ontario, London, ON Canada; 2 Department of Pediatrics, Schulich School of Medicine and Dentistry, University of Western Ontario, London, ON Canada; 3 PERFORM Centre, Concordia University, Montréal, QC Canada; 4 Department of Informative Genetics, Environment and Genome Research Center, Tohoku University Graduate School of Medicine, Sendai, Japan; 5 Department of Oncology, Schulich School of Medicine and Dentistry, University of Western Ontario, London, ON Canada; 6 Children’s Health Research Institute, Lawson Health Research Institute, London, ON Canada

**Keywords:** Cell proliferation, Differentiation, Chromatin remodelling, Epigenetics, Stem-cell differentiation

## Abstract

Cell fusion occurs when several cells combine to form a multinuclear aggregate (syncytium). In human placenta, a syncytialized trophoblast (syncytiotrophoblast) layer forms the primary interface between maternal and fetal tissue, facilitates nutrient and gas exchange, and produces hormones vital for pregnancy. Syncytiotrophoblast development occurs by differentiation of underlying progenitor cells called cytotrophoblasts, which then fuse into the syncytiotrophoblast layer. Differentiation is associated with chromatin remodeling and specific changes in gene expression mediated, at least in part, by histone acetylation. However, the epigenetic regulation of human cytotrophoblast differentiation and fusion is poorly understood. In this study, we found that human syncytiotrophoblast development was associated with deacetylation of multiple core histone residues. Chromatin immunoprecipitation sequencing revealed chromosomal regions that exhibit dynamic alterations in histone H3 acetylation during differentiation. These include regions containing genes classically associated with cytotrophoblast differentiation (*TEAD4*, *TP63*, *OVOL1*, *CGB*), as well as near genes with novel regulatory roles in trophoblast development and function, such as *LHX4* and *SYDE1*. Prevention of histone deacetylation using both pharmacological and genetic approaches inhibited trophoblast fusion, supporting a critical role of this process for trophoblast differentiation. Finally, we identified the histone deacetylases (HDACs) HDAC1 and HDAC2 as the critical mediators driving cytotrophoblast differentiation. Collectively, these findings provide novel insights into the epigenetic mechanisms underlying trophoblast fusion during human placental development.

## Introduction

Cell fusion into a syncytium is the process whereby several cells coalesce to form a multinucleated entity, and features in homeostasis of several normal tissues and pathologies (e.g., cancer, virus infection, inflammation)^1,2^. One of the best paradigms of syncytium formation occurs in the placenta—the organ that forms the interface between a pregnant mother and her baby. The placental exchange surface is lined by a syncytium, called syncytiotrophoblast, which produces hormones and facilitates transfer of nutrients and gases between maternal and fetal blood. Syncytiotrophoblast is formed by fusion of underlying progenitor cells called cytotrophoblasts^3^. Continuous fusion of cytotrophoblasts is crucial for syncytiotrophoblast to expand and replenish, ensuring proper function of the exchange surface throughout pregnancy^4^. Dysregulation of syncytiotrophoblast formation leads to poor placental function, and is associated with several highly prevalent pregnancy complications that are detrimental to maternal and fetal health^5–7^.

Syncytiotrophoblast nuclei exhibit a wide range of distinctive chromatin condensation patterns, with some nuclei demonstrating an open euchromatic appearance and others possessing a more heterochromatic state. Previous studies have indicated that syncytiotrophoblast nuclei are transcriptionally inert based on assessing uptake of [3H]-uridine, which correlates with the high number of heterochromatic nuclei^8,9^. Follow-up studies show that at least some nuclei are transcriptionally active^10^. These studies infer that cytotrophoblast progression into syncytiotrophoblast is associated with altered chromatin dynamics, which may be required for the robust changes in transcription required for syncytiotrophoblast formation and function.

Histone acetylation is an epigenetic modification in which an acetyl group is covalently added to lysine residues on histone tails protruding from nucleosomes, and is a key determinant of chromatin accessibility^11^. The dynamics of histone acetylation in regulating chromatin structure is fundamentally important for the precise timing and level of gene transcription. Histone acetylation is catalyzed by enzymes with acetyltransferase activity, and is associated with open chromatin regions and active transcription. Deacetylation is typically associated with chromatin condensation, and is performed by enzymes called histone deacetylases (HDACs)^12^. Although HDACs are classically associated with inactive transcription, both acetyltransferases and HDACs bind to chromatin near sites of actively transcribed regions, where they function cooperatively to fine-tune histone acetylation and facilitate binding of machinery required for transcription^13^. HDACs are arranged into four distinct classes, of which class I, II, and IV have a zinc-dependent active site that is inhibited by trichostatin A (TSA). Class I HDACs reside in nuclei, and consist of HDAC1, HDAC2, HDAC3, and HDAC8. Class II HDACs shuttle between nucleus and cytoplasm, and include HDAC4, HDAC5, HDAC6, HDAC7, HDAC9, and HDAC10. HDAC11 is the sole member of class IV^14^. Class III HDACs are a distinct family identified as sirtuins, which depend on nicotinamide adenine dinucleotide for deacetylase activity^15^.

Due to their critical role as regulators of chromatin accessibility and gene transcription, histone acetylation dynamics serve a crucial role in the control of cell proliferation and differentiation^16^. For example, HDAC1 regulates embryonic stem cell differentiation^17,18^, and controls lineage-specific transcriptional networks in mouse embryonic and trophoblast stem cells^19^. Blocking HDAC activity in mouse trophoblast stem cells promotes labyrinth trophoblast development at the expense of giant cell and spongiotrophoblast formation^20^. Class II HDACs are important for proper trophoblast differentiation in mouse placentas^21^, and mouse trophoblast stem cells lacking Sirtuin-1 show blunted cell differentiation^22^. In human trophoblasts, HDAC inhibition impairs expression of the efflux protein P-glycoprotein, and the cortisol metabolizing enzyme 11β-hydroxysteroid dehydrogenase^23,24^. Recent derivation of human trophoblast stem cells was made possible, in part, by using an HDAC inhibitor to maintain cells in a stem state, suggesting a critical role for HDAC activity in human trophoblast differentiation^25^. Indeed, differentiation of primary human cytotrophoblasts is associated with profound changes in histone acetylation, including gene- and promoter-specific changes in H3K9 and H3K27 acetylation^26^. Collectively, these studies emphasize the critical role of histone acetylation dynamics and HDAC activity in the regulation of trophoblast differentiation and placental development, which prompted us to investigate the functional role of specific HDACs during human syncytiotrophoblast formation.

In this study, we found that histone acetylation decreases as trophoblast cells and cell lines differentiate into syncytiotrophoblast, and that HDAC1 and HDAC2 activity is required for cytotrophoblast differentiation. We identified several genomic regions in which acetylation patterns are altered during cytotrophoblast differentiation that correlate with robust changes in gene transcription required for differentiation. Collectively, our results reveal a critical role of HDACs for the regulation of cytotrophoblast differentiation, and provide new insights into the epigenetic regulation of human syncytiotrophoblast formation.

## Results

### Expression of acetylated histone proteins in human placenta and differentiating cytotrophoblasts

**Fig. 1:**
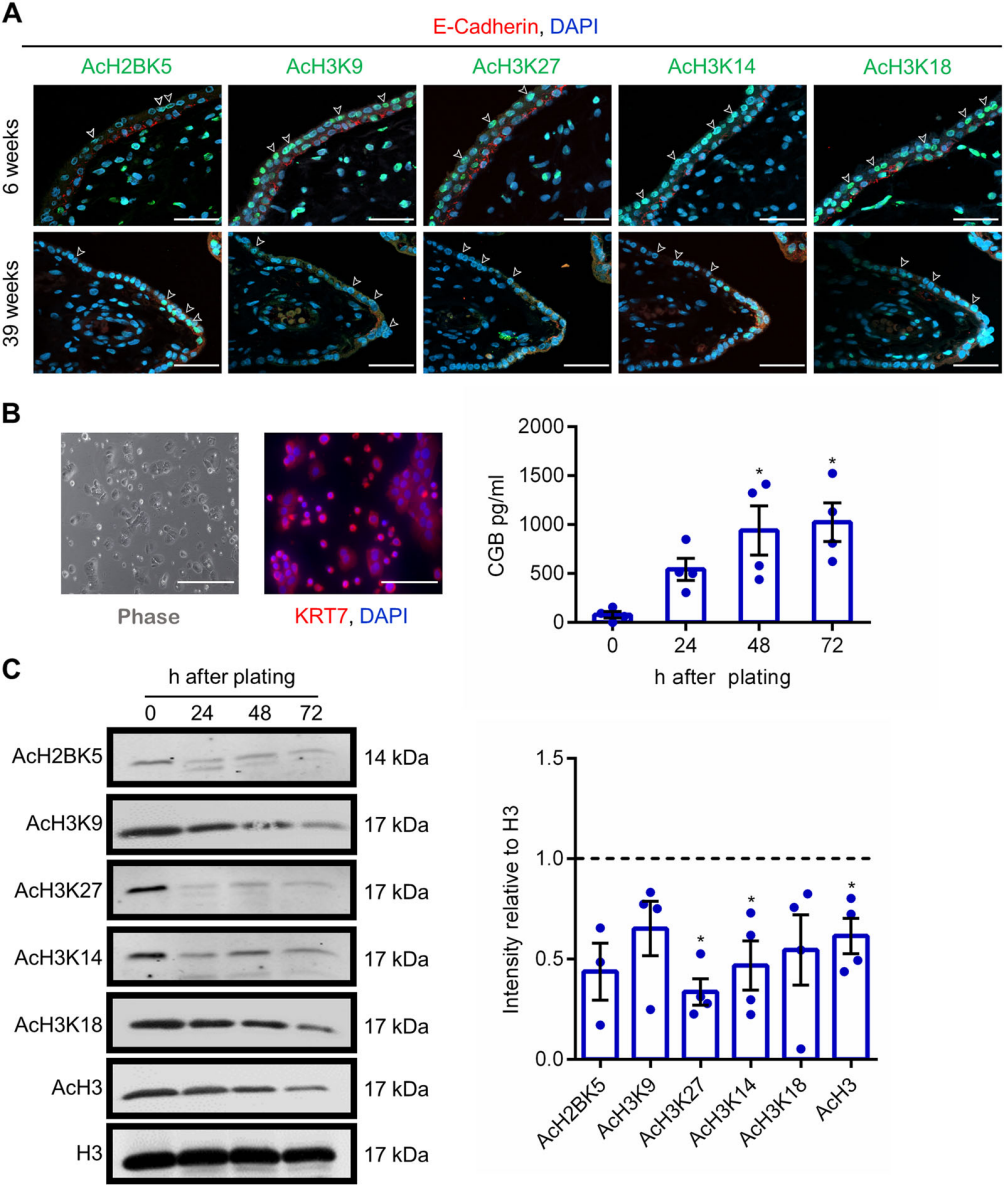
Histone acetylation patterns in human placenta and primary cytotrophoblasts. **a** Localization of AcH2BK5, AcH3K9, AcH3K27, AcH3K14, and AcH3K18 in 6-week and 39-week human placenta. In all panels, cytotrophoblasts were detected by immunostaining for E-cadherin (red), and nuclei were counterstained using DAPI (blue). Arrowheads denote the syncytiotrophoblast layer. **b** Primary cytotrophoblasts were isolated from term human placenta, and cultured for 24, 48, and 72 h. At the 24-h timepoint, KRT7 (red) was detected by immunofluorescence to validate that these cells were trophoblasts. Nuclei were counterstained using DAPI (blue). CGB production was determined in conditioned media by enzyme immunoassay. **c** Levels of AcH2BK5, AcH3K9, AcH3K27, AcH3K14, AcH3K18, AcH3, and total histone H3 (loading control) were determined by western blotting (*n* = 4). Densitometric analysis relative to total histone H3 is shown beside the representative western blots. The dotted line represents signal intensity in undifferentiated cells. Graphs represent means ± SEM. Data significantly different from undifferentiated cells are indicated by an asterisk (*P < 0.05; *n* = 4 from different placentas). Scale bars represent 40 μm.

To evaluate localization of acetylated histone proteins in human placenta, we performed immunohistochemistry to detect acetylated histone H2B K5 (AcH2BK5), acetylated histone H3 (AcH3) K9, AcH3K27, AcH3K14, and AcH3K18 in 6-week and 39-week human placenta. All acetylated histone lysine residues were identified in both cytotrophoblasts (identified using E-cadherin) and syncytiotrophoblast in 6-week placentas (Fig. 1a). Acetylated histones were also detected in the villous core. In 39-week placentas, few cytotrophoblasts were visible, and staining intensity of acetylated histone proteins in syncytiotrophoblast appeared to be lower than in early gestation placentas. Interestingly, patterns of histone acetylation were not consistent between cells of the same lineage, with some cells exhibiting robust expression of histone acetylation and other cells with low-to-undetectable expression. These findings indicate that histone acetylation patterns in the human placenta are dynamic, and may be associated with altered function or developmental progression within placental cells. Since images represent a snapshot of the human placenta and we cannot confirm the developmental stage of each cell, we sought to analyze histone acetylation patterns in primary cytotrophoblasts, where cells exhibit synchrony in terms of their developmental progression.

Immunofluorescence for cytokeratin-7 was used to evaluate the purity of the isolated cytotrophoblast populations (Fig. 1b). Cytotrophoblasts were then lysed immediately before plating (0 h), or cultured for 24, 48, or 72 h, during which time they spontaneously differentiate into syncytiotrophoblast and produce high levels of the syncytiotrophoblast hormone subunit chorionic gonadotropin beta (CGB) (Fig. 1b). Interestingly, levels of AcH3K27, AcH3K14, and AcH3 significantly decreased at 72 h differentiation (Fig. 1c). Levels of AcH2BK5, AcH3K9, and AcH3K18 appeared to be decreased, but did not reach statistical significance (*P* = 0.058, *P* = 0.082, and *P* = 0.080, respectively).

**Fig. 2:**
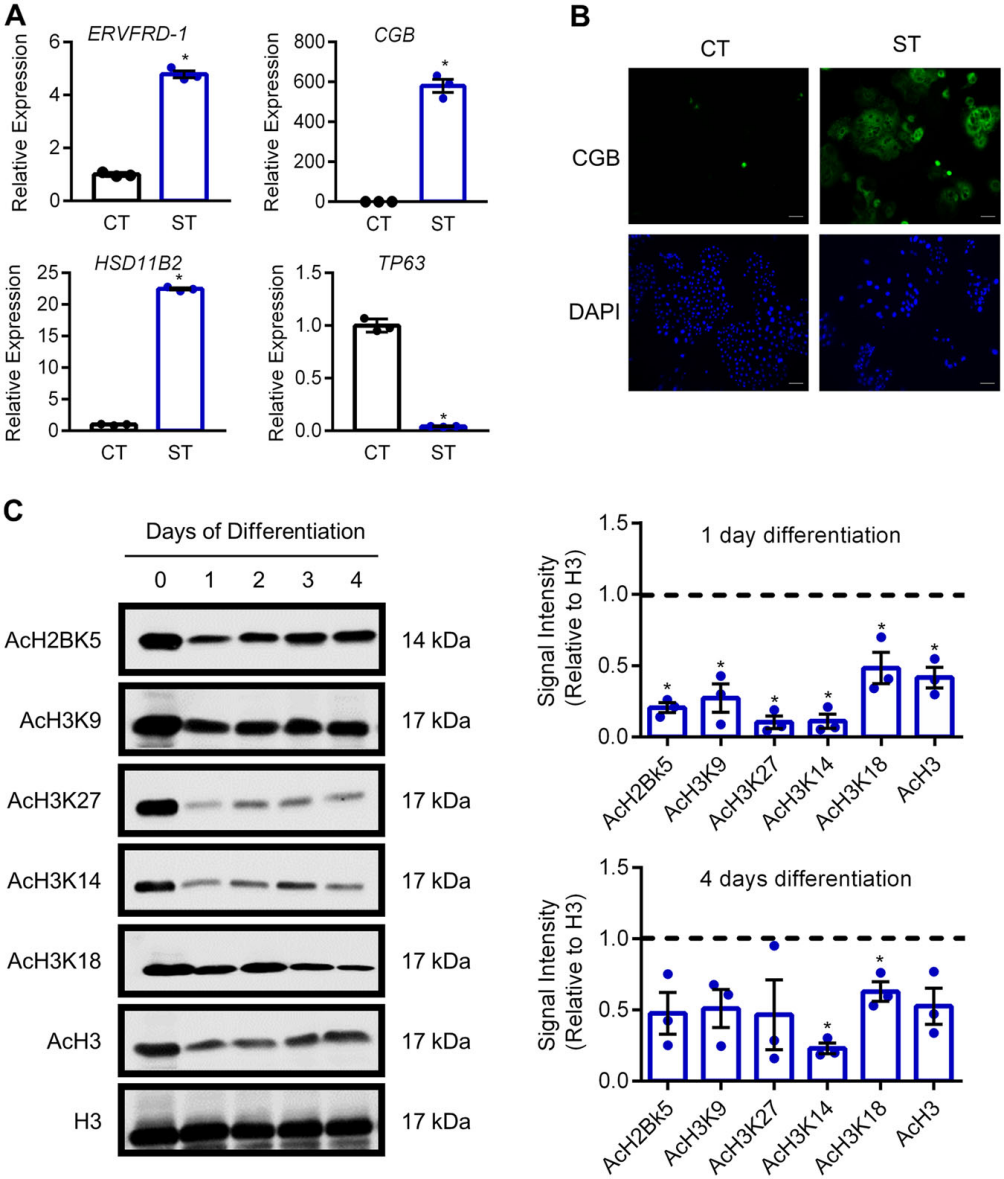
Reduced histone acetylation during syncytialization of human trophoblast stem cells. **a** Transcript levels of *ERVFRD-1*, *CGB*, *HSD11B2*, and *TP63* in human trophoblast stem cells cultured in stem conditions, and following 5 days culture in conditions that promote syncytiotrophoblast formation. **b** Detection of CGB in human trophoblast stem cells cultured in stem and differentiation conditions. Nuclei were counterstained using DAPI (blue). **c** Levels of AcH2BK5, AcH3K9, AcH3K27, AcH3K14, AcH3K18, AcH3, and total H3 were determined by western blotting. Densitometric analysis relative to total histone H3 on day 1 and day 4 of syncytialization is shown to the right of the western blots. The dotted line represents signal intensity in undifferentiated cells. Graphs represent means ± SEM. Data significantly different from undifferentiated cells are indicated by an asterisk (*P* < 0.05; *n* = 3). Scale bars represent 100 μm.

Next, we investigated patterns of histone acetylation in recently-derived human trophoblast stem cells maintained in the stem state, or following differentiation into syncytiotrophoblast. When induced to differentiate toward the syncytiotrophoblast lineage, human trophoblast stem cells exhibit increased expression of *ERVFRD-1* (4.7-fold, encodes syncytin-2), *CGB* (579.9-fold), and *HSD11B2* (22.4-fold, encodes the glucocorticoid-inactivating enzyme corticosteroid 11-β-dehydrogenase isozyme 2), and decreased expression of *TP63* (17-fold; all *P* < 0.05, Fig. 2a). CGB is also highly detectable at the protein level (Fig. 2b). Not surprisingly, during TS cell differentiation toward syncytiotrophoblast, removal of the HDAC inhibitor valproic acid (used as a media component to maintain these cells in a stem state) correlated with reduced levels of AcH2BK5, AcH3K9, AcH3K27, AcH3K14, AcH3K18, and AcH3 as early as one day after onset of differentiation (all *P* < 0.05, Fig. 2c). The reduced acetylation during syncytialization is consistent with our observations in primary cytotrophoblasts. After four days of syncytiotrophoblast development, levels of histone acetylation remained lower than in the undifferentiated state, although only AcH3K14 and AcH3K18 were statistically significant.

**Fig. 3:**
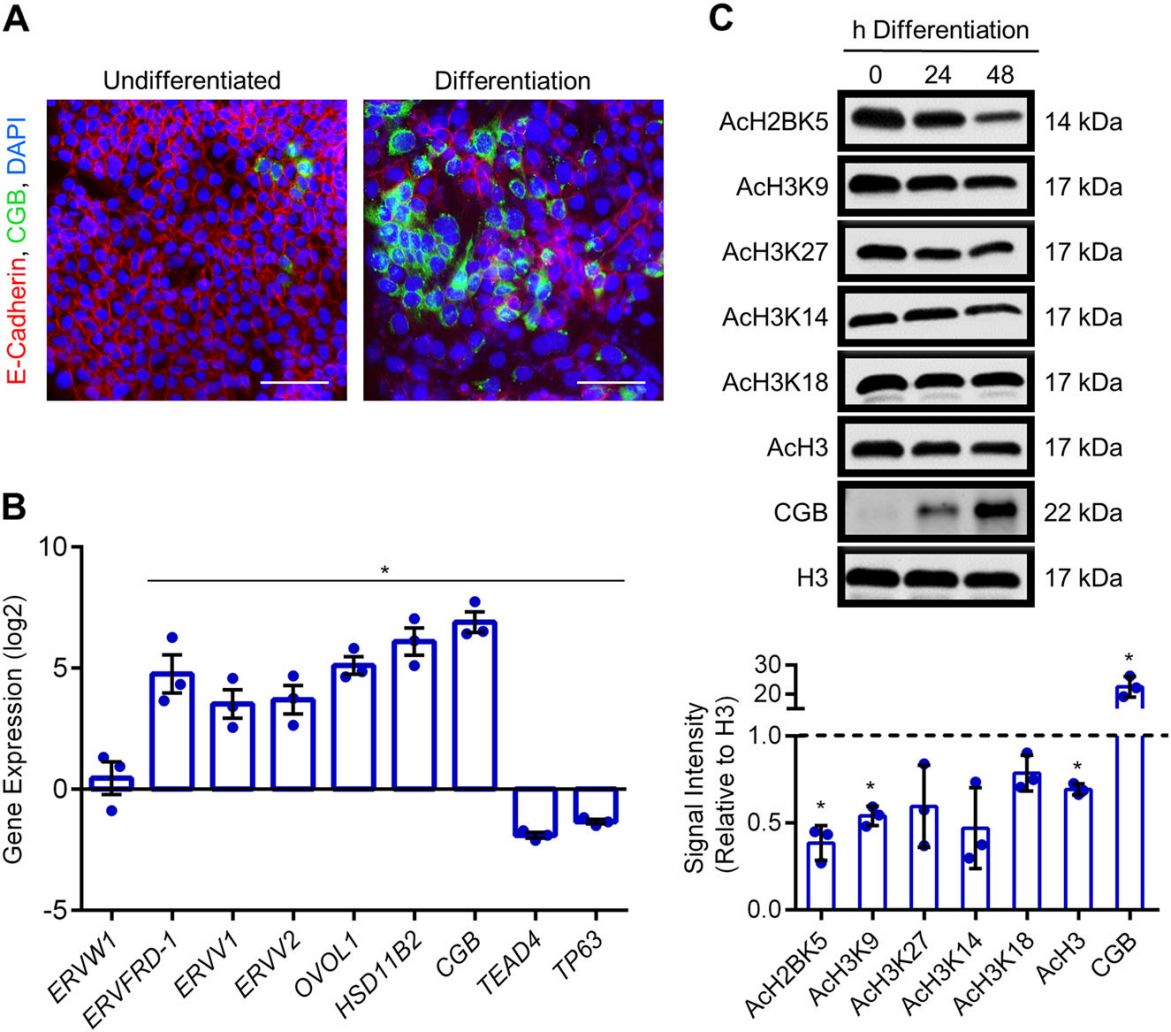
Reduced histone acetylation during syncytialization of BeWo trophoblasts. **a** Representative immunofluorescent images depicting E-cadherin and CGB expression in BeWo trophoblasts cultured in undifferentiated and differentiation conditions. Nuclei were counterstained using DAPI (blue). Please note the loss of E-cadherin in syncytialized cells, and increased production of CGB. **b** Transcript levels of various genes associated with cytotrophoblast progenitor traits (*TP63*, *TEAD4*) and syncytiotrophoblast development (*ERVW1, ERVFRD-1, ERVV1, ERVV2, OVOL1, HSD11B2*, and *CGB*) in cells cultured in undifferentiated and differentiation conditions. **c** Levels of AcH2BK5, AcH3K9, AcH3K27, AcH3K14, AcH3K18, AcH3, CGB, and total H3 were determined by western blotting. Densitometric analysis relative to total histone H3 is shown below the representative western blots. The dotted line represents signal intensity in undifferentiated cells. Graphs represent means ± SEM. Data significantly different from undifferentiated cells are indicated by an asterisk (*P < 0.05; *n* = 3). Scale bars represent 80 μm.

To further examine histone acetylation patterns during cytotrophoblast differentiation, we used BeWo trophoblasts—a transformed cytotrophoblast cell line that is commonly used to study dynamics of cytotrophoblast differentiation into syncytiotrophoblast. The advantage of using these cells is that onset of differentiation can be precisely timed by exposing cells to a cell-permeable derivative of 3′, 5′-cyclic adenosine monophosphate (8-Br-cAMP), resulting in decreased surface expression of junctional proteins such as E-cadherin and increased production of CGB (Fig. 3a). Differentiation of BeWo trophoblasts was associated with increased mRNA expression of *CGB* (130.5-fold, *P* < 0.05)*, HSD11B2* (78.9-fold, *P* < 0.05), *ERVW1* (1.6-fold, encodes syncytin-1), *ERVFRD-1* (36.7-fold, *P* < 0.05), the retroviral genes *ERVV1* and *ERVV2* (13.4-fold and 15.1-fold, respectively, both *P* < 0.05), and *OVOL1* (36.8-fold, encodes a transcriptional repressor essential for cytotrophoblast differentiation, *P* < 0.05; Fig. 3b). Differentiation was also associated with decreased expression of genes encoding transcription factors associated with cytotrophoblast progenitor traits (*TP63*: 2.6-fold; *TEAD4*: 3.8-fold; Fig. 3b, *P* < 0.05). At the protein level, differentiation was associated with a 22.5-fold upregulation of CGB, and reduced levels of AcH2BK5, AcH3K9, and AcH3 (62%, 46%, and 31% decreased at 48 h differentiation compared with undifferentiated cells, respectively; Fig. 3c, all *P* < 0.05). Levels of AcH3K27, AcH3K14, and AcH3K18 appeared to be reduced in differentiated cells, but did not reach statistical significance (*P* = 0.22, *P* = 0.058, and *P* = 0.13, respectively). Thus, trophoblast differentiation into syncytiotrophoblast is associated with reduced acetylation of multiple histone lysine residues.

### Dynamic changes in genomic AcH3 binding during syncytiotrophoblast development

**Fig. 4:**
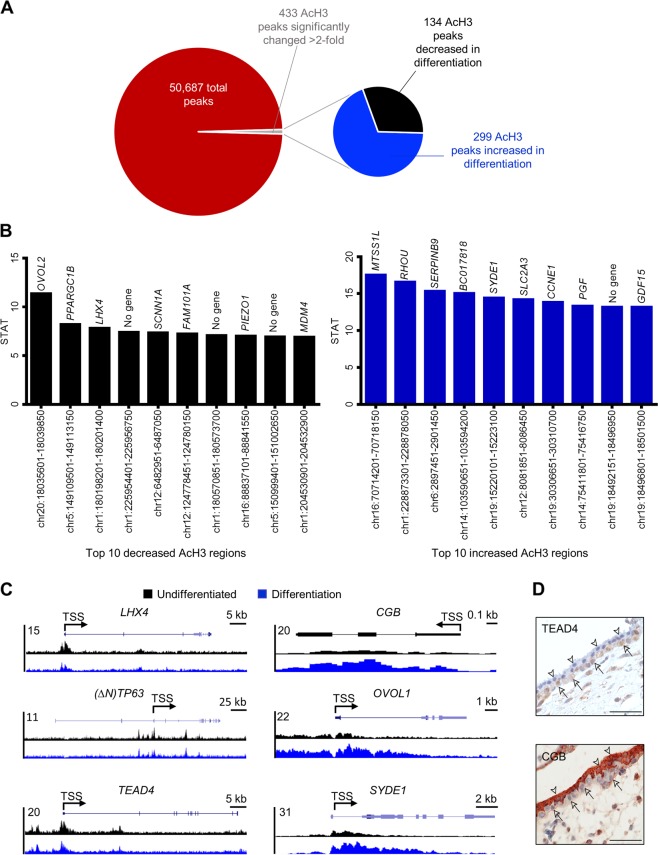
Dynamic changes in genomic AcH3 binding during trophoblast differentiation. **a** Pie chart showing total number of AcH3 peaks, and the number of peaks that increased or decreased AcH3 binding during syncytiotrophoblast development by at least twofold (*P*_adj_ < 0.01). **b** Top 10 chromosomal regions exhibiting decreased (black bars) and increased (blue bars) AcH3 binding during differentiation. Genes located proximate (<1 kb) to these chromosomal areas are included above each bar. **c** Representative genome browser views of chromosomal regions proximate to *LHX4*, (*ΔN*)*TP63*, *TEAD4*, *CGB*, *OVOL1*, and *SYDE1*, which exhibit differential AcH3 binding in undifferentiated and differentiation conditions. **d** Immunohistochemistry for TEAD4 and CGB on first trimester human placental sections. Arrowheads denote syncytiotrophoblast; arrows point to cytotrophoblasts. Scale bars represent 50 μm.

Since syncytiotrophoblast development was associated with reduced histone acetylation, our next goal was to use chromatin immunoprecipitation sequencing to evaluate site-specific changes in histone H3 acetylation in undifferentiated BeWo trophoblasts versus cells undergoing differentiation. We identified 50,678 AcH3 peaks genome wide, of which 433 peaks were significantly changed at least twofold (*P*_adj_ < 0.01) between undifferentiated and differentiated cells. One hundred and thirty-four peaks (30.9%) exhibited at least twofold higher H3 acetylation in undifferentiated cells, whereas higher H3 acetylation in differentiation conditions was detected at 299 peaks (69.1%, Fig. 4a). The top ten chromosomal regions exhibiting higher AcH3 occupancy in undifferentiated cells and differentiated cells are shown in Fig. 4b. Interestingly, ten of the top twenty peaks that exhibited robust deacetylation during differentiation aligned to chromosomes 1 and 5, whereas eight of the top twenty peaks showing increased acetylation were located on chromosomes 14 and 19, which is consistent with a large number of genes on these chromosomes expressed in syncytiotrophoblast^27^. Increased AcH3 binding in undifferentiated cells was also identified near genes previously associated with cytotrophoblast stem traits (*TEAD4* at chr12:3066251-3068900, *TP63* at chr3:189507601-189509350, Fig. 4c, both *P* < 0.01; TEAD4 localized to cytotrophoblasts in human placenta is shown in Fig. 4d). Increased AcH3 in differentiated cells was consistently detected close to genes associated with syncytiotrophoblast development and function (*CGB* at chr19:49525501-49525850, *OVOL1* at chr11:65554451-65557400, *ERVW1* at chr7:92098079-92099695, *ERVFRD-1* at chr6:11102722-11112071, *HSD11B2* at chr16:67465036-67471454, Fig. 4c and Fig. S1, all *P* < 0.01; CGB localized to syncytiotrophoblast in human placenta is shown in Fig. 4d). Additionally, we also discovered several regions that exhibit differential AcH3 levels located near genes that have not been previously associated with human syncytiotrophoblast development. These novel targets include a differentiation-associated increase in AcH3 near the gene encoding synapse defective 1 (*SYDE1*), which is a Rho-GTPase activating protein stimulated by glial cells missing-1, and is important for murine placentation and human trophoblast migration^28^. Decreased AcH3 binding was detected during differentiation near LIM homeobox 4 (*LHX4*), which is important for development of the placental labyrinth zone in mice^29^.

### HDAC inhibition prevents cytotrophoblast differentiation

**Fig. 5:**
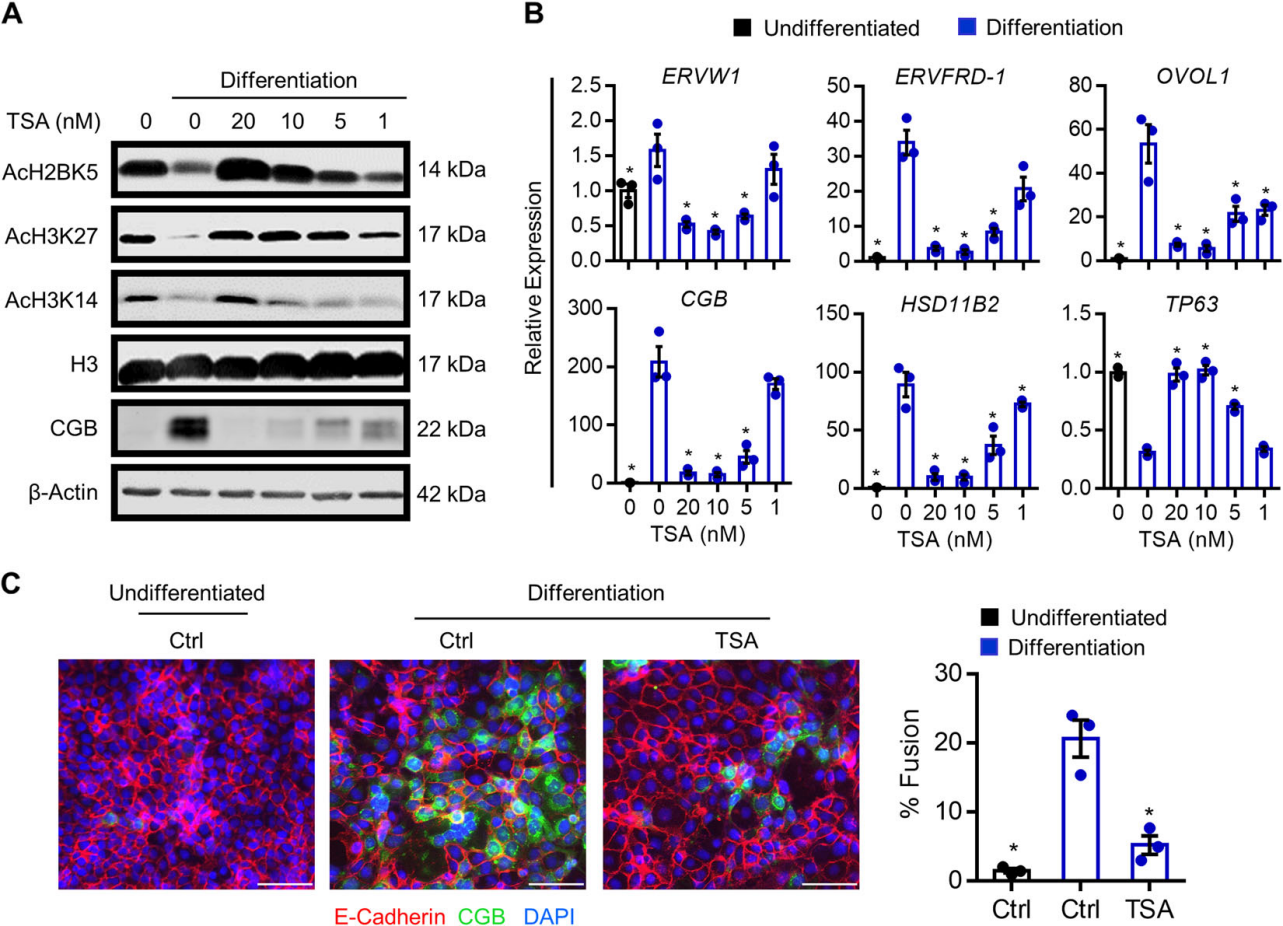
Effect of HDAC inhibition on histone acetylation and syncytiotrophoblast development. BeWo trophoblasts were exposed to the broad spectrum HDAC inhibitor trichostatin A (TSA, 0–20 nM), and then induced to differentiate for 48 h. **a** Levels of AcH2BK5, AcH3K27, AcH3K14, histone H3, CGB, and β-Actin (loading control) were determined by western blotting. **b** Transcript levels of *ERVW1, ERVFRD-1*, *OVOL1*, *CGB*, *HSD11B2*, and *TP63* in cells cultured in undifferentiated (black bar) and differentiation (blue bars) conditions with or without various doses of TSA. **c** Percentage of fused cells following culture in undifferentiated or differentiation conditions with or without 20 nM TSA. Representative images of E-cadherin (red) and CGB (green) are shown to the left of the graph. Nuclei were counterstained using DAPI (blue). Scale bar represents 80 μm. Graphs represent means ± SEM. Data significantly different from the Ctrl (0 nM TSA) cells cultured in differentiation conditions are indicated by an asterisk (*P < 0.05; *n* = 3 in (**b**), *n* = 9 images from three experiments in (**c**)).

Altered acetylation levels of multiple histone residues during cytotrophoblast differentiation suggest that HDACs may be important mediators of syncytiotrophoblast development. To determine the impact of HDAC activity on trophoblast differentiation, BeWo trophoblasts were treated with the broad spectrum HDAC inhibitor, TSA, and then induced to differentiate for up to 48 h. TSA dose-dependently increased levels of AcH2BK5, AcH3K27, and AcH3K14 (Fig. 5a). To determine the impact of TSA on transcripts associated with cytotrophoblast differentiation, we assessed expression of genes that change during differentiation (Fig. 5b). Exposure of cells to TSA prior to commencing differentiation dose-dependently inhibited expression of all genes that are normally induced during differentiation, including *ERVW1*, *ERVFRD-1, OVOL1*, *CGB*, and *HSD11B2* (Fig. 5b, *P* < 0.05). In the presence of 10 and 20 nM TSA, expression of *TP63* was maintained at levels comparable to undifferentiated cells (Fig. 5b). Furthermore, compared with cells exposed to differentiation conditions for 48 h, in which nuclei were frequently contained within fused cell clusters (E-cadherin-negative, CGB-positive), 74.9% less nuclei were detected within fused clusters in the presence of 20 nM TSA (*P* < 0.05, Fig. 5c). Thus, inhibition of HDAC activity prevented syncytiotrophoblast formation.

To test the efficacy of another broad-spectrum inhibitor of HDAC activity on syncytiotrophoblast development, BeWo trophoblasts were treated with various doses of SAHA. Cells exposed to SAHA under differentiation conditions exhibited a similar prevention of histone deacetylation and dose-dependent restoration of transcript expression comparable to levels detected in undifferentiated cells, as was observed with TSA (Fig. S2). Likewise, exposure of cells to SAHA prevented syncytiotrophoblast development by 72.7% (Fig. S2C, *P* < 0.05). Since both TSA and SAHA abrogated cytotrophoblast differentiation, we conclude that HDAC activity is required for syncytiotrophoblast development.

### HDAC1 and HDAC2 are critical regulators of cytotrophoblast differentiation

**Fig. 6:**
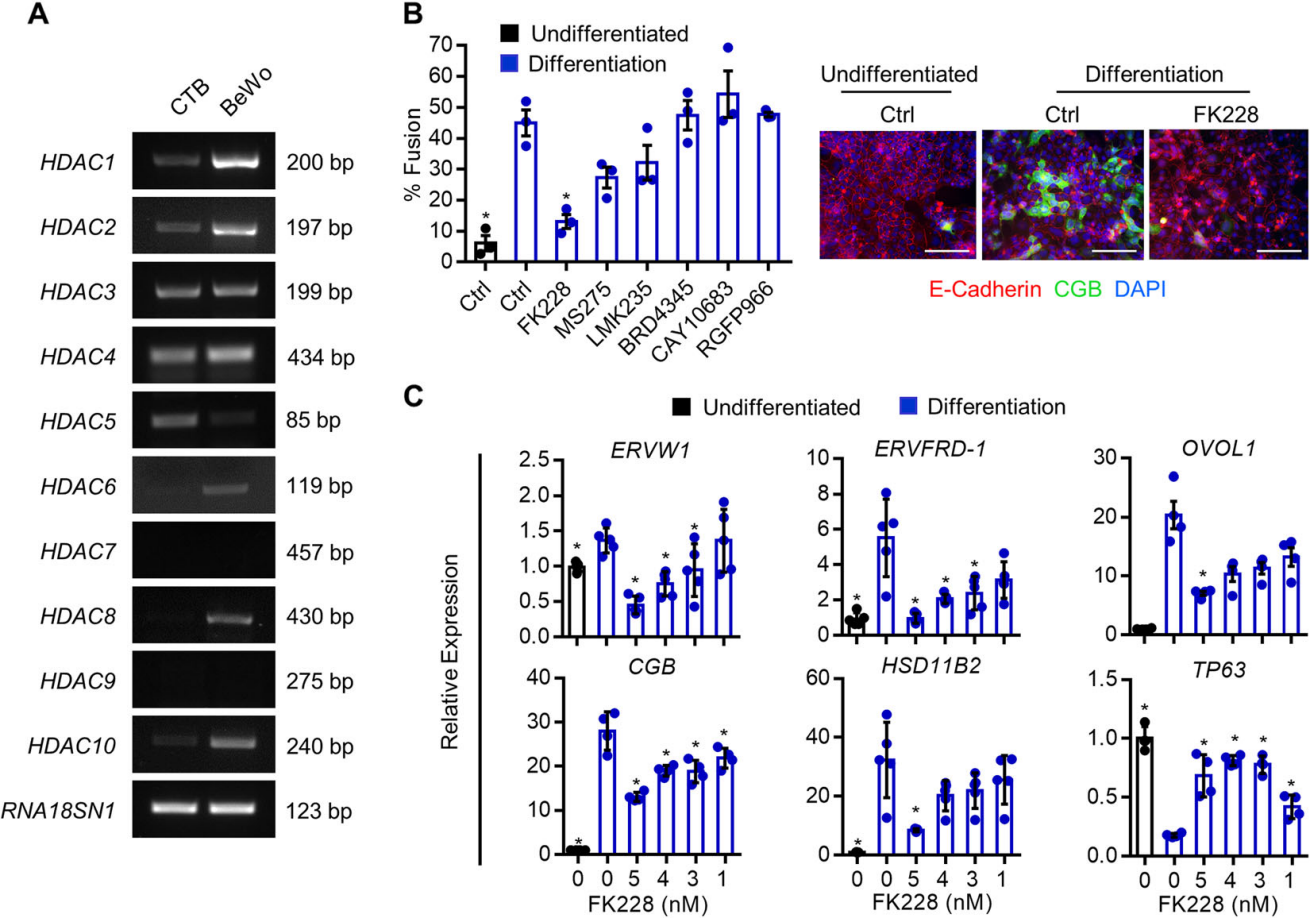
Effect of selective HDAC inhibitors on syncytiotrophoblast development. **a** RT-PCR depicting expression of *HDAC1-10* in primary term cytotrophoblasts (CTB) and BeWo trophoblasts. *RNA18SN1* was used to ensure that a similar quantity of cDNA was used in PCR reactions. **b** Percentage of BeWo trophoblasts that underwent syncytialization following culture in undifferentiated conditions (black bar), or differentiation conditions (blue bars) in the presence of FK228, MS275, LMK235, BRD4345, CAY10683, or RGFP966. Please note that only the HDAC1/HDAC2 inhibitor FK228 significantly inhibited cytotrophoblast differentiation. Representative images of E-cadherin (red) and CGB (green) in undifferentiated and differentiation conditions with or without 5 nM FK228 is shown to the right of the graph. Nuclei were counterstained using DAPI (blue). Scale bar represents 80 μm. **c** Transcript levels of *ERVW1, ERVFRD-1*, *OVOL1*, *CGB*, *HSD11B2*, and *TP63* in cells cultured in undifferentiated and differentiation conditions with or without various doses of FK228. Graphs represent means ± SEM. Data significantly different from control cells cultured in differentiation conditions are indicated by an asterisk (*P < 0.05; *n* = 9 images from three experiments in (**b**), *n* = at least 4 in (**c**)).

We next investigated the role of specific HDACs in cytotrophoblast differentiation. First, we profiled expression of HDAC1 to HDAC10 in human primary cytotrophoblasts and BeWo trophoblasts (Fig. 6a). *HDAC1*, *HDAC2*, *HDAC3*, *HDAC4*, *HDAC5*, and *HDAC10* were detectable in both primary cytotrophoblasts and BeWo trophoblasts. *HDAC6* and *HDAC8* were detectable in BeWo trophoblasts, but expression was low or undetectable in primary cytotrophoblasts. HDAC7 and HDAC9 were not detectable in either cell-type. To narrow our search for specific HDACs involved in syncytiotrophoblast development, cells cultured under differentiation conditions were treated with small molecule inhibitors that decrease activity of defined subsets of HDACs. Dose and specificity of the HDAC inhibitors used in this study are provided in Table 1. The only HDAC inhibitor that prevented differentiation was the HDAC1/HDAC2 inhibitor FK228 (71% decrease in fusion, *P* < 0.05, Fig. 6b). The highest concentration of FK228 (5 nM) inhibited expression of the differentiation-associated transcripts *ERVW1* (66.9%), *ERVFRD*-1 (82.6%), *OVOL1* (68.6%), *CGB* (51.9%), and *HSD11B2* (73.9%, all *P* < 0.05), and prevented differentiation-associated repression of *TP63* by 75% (Fig. 6c, *P* < 0.05).

**Table 1:** HDAC inhibitors used in this study.

Name	Specificity	Dose	Company, catalog
BRD4345^66^	HDAC5, HDCA9	1300 nM	Sigma-Aldrich SML1706
CAY10683^67^	HDAC2	300 nM	Cayman Chemical Co 15403-5
FK228^68^	HDAC1, HDAC2	5 nM	Sigma-Aldrich SML1175
LMK235^69^	HDAC4, HDAC5	880 nM	Sigma-Aldrich SML1053
MS275^70^	HDAC1, HDAC3	740 nM	Cayman Chemical Co 13284-1
RGFP966^71^	HDAC3	160 nM	Cayman Chemical Co 16917-1
SAHA^72^	Pan	50-750 nM	Sigma-Aldrich SML0061
TSA^73^	Pan	1–20 nM	Sigma-Aldrich T8552

To further determine the biological significance of HDAC1 in syncytiotrophoblast development, we delivered two-distinct shRNAs into BeWo trophoblasts, and generated stable populations of cells with constitutively decreased HDAC1 expression (HDAC1-KD1 and HDAC1-KD2). Using this strategy, *HDAC1* mRNA was decreased by 87.5% in HDAC1-KD1 cells, and 64.6% in HDAC1-KD2 cells compared with controls (*P* < 0.05, Fig. S3A). HDAC1 protein was also substantially reduced in both KD1 and KD2 cells (Fig. S3B). There was no significant effect of HDAC1 knockdown on mRNA expression of *HDAC2*, but an increase in HDAC2 protein was apparent, which is consistent with findings reported in other cells in which HDAC1 is reduced or absent^30^. There was no significant effect of HDAC1 deficiency on the ability of cells to differentiate (Fig. S3D), nor was there any difference in transcript expression of *ERVFRD-1*, *HSD11B2*, and *TP63* (Fig. S3C). A modest reduction of *CGB* expression was observed in HDAC1-KD2 cells (54.4%, *P* < 0.05, Fig. S3C), but not in HDAC1-KD1 cells.

We employed a similar strategy to decrease HDAC2 expression. Stable populations of BeWo trophoblasts expressing shRNAs targeting HDAC2 exhibited reduced mRNA levels of *HDAC2* (90.5% and 87.6% decreased in HDAC2-KD1 and HDAC2-KD2 cells, respectively, *P* < 0.05, Fig. S4A), and showed reduced HDAC2 protein (Fig. S4B). Cells lacking HDAC2 were able to robustly differentiate (Fig. S4D), and there were no consistent differences in the expression of differentiation-associated genes in HDAC2-KD1 and HDAC2-KD2 cells compared with control cells (Fig. 4C). Thus, deficiency of HDAC1 or HDAC2 is not sufficient to prevent syncytiotrophoblast development.

**Fig. 7:**
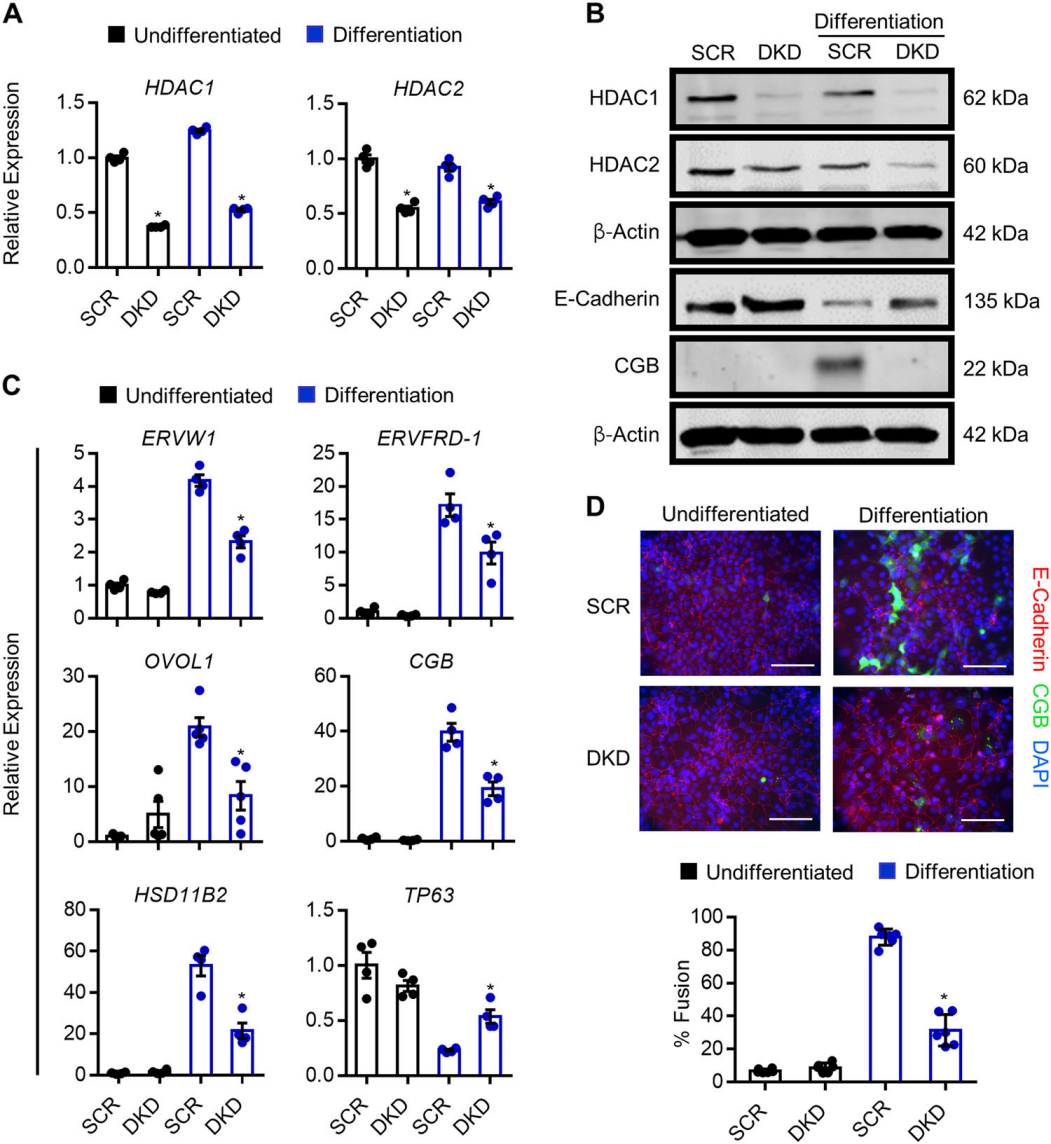
Knockdown of HDAC1 and HDAC2 inhibits syncytiotrophoblast development. **a** Expression of *HDAC1* and *HDAC2* in BeWo trophoblasts expressing control (scrambled, SCR) shRNA or shRNAs targeting HDAC1 and HDAC2 (double knockdown, DKD) cultured in undifferentiated (black bars) and differentiation (blue bars) conditions. **b** Western blot showing protein levels of HDAC1, HDAC2, E-cadherin, and CGB in SCR and DKD cells cultured in undifferentiated and differentiation conditions. β-Actin shown in the third blot (from top) served as a loading control for HDAC1 and HDAC2 blots; β-Actin shown in the sixth blot served as a loading control for E-cadherin and CGB. **c** Transcript levels of *ERVW1, ERVFRD-1*, *OVOL1*, *CGB, HSD11B2*, and *TP63* in SCR and DKD cells cultured in undifferentiated and differentiation conditions. **d** Percentage of SCR and DKD cells that underwent syncytialization (E-cadherin-negative, CGB-positive) following culture in undifferentiated or differentiation conditions. Representative images of E-cadherin (red) and CGB (green) is shown above the graph. Nuclei were counterstained using DAPI (blue). Graphs represent means ± SEM. Data significantly different from SCR cells cultured in differentiation conditions are indicated by an asterisk (*P < 0.05; *n* = at least 4 in (**a**) and (**c**), *n* = 18 images from six experiments in (**d**). In (**c**) and (**d**), statistical comparisons are shown only for differentiation conditions). Scale bars represent 80 μm.

Since HDAC1 and HDAC2 are highly similar proteins that have the potential to compensate for each other^31^, we next analyzed the impact of reduced expression of both HDAC1 and HDAC2 on trophoblast differentiation capacity. Stable populations of HDAC1-KD1 cells were transduced with lentivirus carrying HDAC2-KD1 shRNAs, resulting in cells that exhibited a 59.6% decrease in *HDAC1* expression and 34.7% decrease in *HDAC2* expression (*P* < 0.05, Fig. 7a). Since expression of both HDAC1 and HDAC2 was reduced, these cells are henceforth referred to as “double knockdown” (DKD) cells. DKD cells exhibited a mild proliferation defect under undifferentiated conditions (not shown), which may explain the reduced knockdown efficiency of *HDAC1* and *HDAC2* in DKD cells, since some HDAC1/HDAC2 expression is likely required in the undifferentiated state. Under differentiation conditions, DKD cells exhibited impaired differentiation capacity, including reduced protein expression of CGB and increased E-cadherin levels (Fig. 7b), and a 64.2% decrease in the number of cells that underwent fusion (*P* < 0.05, Fig. 7d). Furthermore, compared with control cells subjected to differentiation conditions, DKD cells exhibited significantly reduced expression of *ERVW1* (45%)*, ERVFRD-1* (45.8%)*, OVOL1* (60%)*, CGB* (51%) and *HSD11B2* (58%), and retained 2.3-fold higher expression of *TP63* (all *P* < 0.05, Fig. 7c). Chromatin immunoprecipitation analysis showed that, when cells were exposed to differentiation conditions, DKD cells had reduced AcH3 enrichment proximate to the transcription start site of both *HSD11B2* and *OVOL1* compared with control cells (Fig. S5). Collectively, these findings indicate that DKD cells have a reduced capacity to differentiate, indicating that HDAC1 and HDAC2 are critical mediators of syncytiotrophoblast development.

## Discussion

In the present study, we found that global acetylation of core histones is altered during progression of cytotrophoblast differentiation into syncytiotrophoblast. We also identified dynamic changes in histone acetylation during trophoblast differentiation, including site-specific changes at loci that require transcriptional regulation during differentiation, and showed that HDAC activity is critical for syncytiotrophoblast formation. Collectively, our discoveries provide novel insights into the epigenetic mechanisms of syncytiotrophoblast development.

Reversible acetylation of specific lysine residues in histone proteins is a major component of the epigenetic code that regulates chromatin accessibility. The acetylation status of histones is controlled through the competing actions of HDACs and histone acetyltransferases, which have a pivotal role in controlling gene expression during cell homeostasis, proliferation, and differentiation. In the current study, we found that coordination of cytotrophoblast differentiation into syncytiotrophoblast was associated with reduced acetylation of multiple histone lysine residues. Syncytialization is associated with a multitude of transcriptional changes^32^, so it was surprising to observe a robust decrease in histone acetylation during the progression of differentiation. One potential explanation is that histone hypoacetylation is required to transiently exit the epithelial state in order to facilitate syncytialization. High levels of histone acetylation are associated with maintenance of the epithelial phenotype^33^, and histone deacetylation is implicated with repression of epithelial characteristics and transition to a mesenchymal state^34,35^. While there is no evidence that syncytiotrophoblast formation requires a mesenchymal transformation, syncytialization necessitates repression of epithelial junctional proteins such as E-cadherin. In line with our study, differentiation of mouse trophoblast stem cells into (predominantly) trophoblast giant cells is associated with decreased acetylation of H2AK5, H2BK5, H2BK12, H2BK15, H2BK20, H3K9, and H4K8, as well as loss of E-cadherin^36^. Thus, histone deacetylation may be a conserved feature of trophoblast differentiation.

Since HDACs catalyze histone deacetylation, we next sought to determine the importance of HDAC activity for syncytialization. We found that the pan-HDAC inhibitors TSA and SAHA, and the selective HDAC1/HDAC2 inhibitor FK228, all robustly inhibited cytotrophoblast differentiation, implicating HDAC1/HDAC2 activity as a critical determinant of syncytiotrophoblast development. HDAC1 and HDAC2 exhibit 86% amino acid sequence identity, and are recruited to appropriate sites by binding to transcription factors as homodimers or heterodimers, or being part of multi-component complexes^37–39^. Their ubiquitous expression, incorporation into similar recruitment complexes, deacetylase activity toward common substrates, and high homology all suggest that HDAC1 and HDAC2 are largely redundant and can compensate for a functional loss of the other. Indeed, mice exhibiting tissue-specific inactivation of *Hdac1* or *Hdac2* in epidermis^17^, B cells^40^, T cells^41^, cardiomyocytes^31^, endothelial cells^42^, neuronal precursors^43^, neural crest^44^, smooth muscle^45^, and oligodendrocytes^46^, all do not exhibit an obvious phenotypic defect, whereas loss of both *Hdac1* and *Hdac2* results in severe phenotypes in these tissues^47^. However, in mice, global deletion of *Hdac1* results in embryonic lethality at midgestation^48^, which is an effect not observed in mice lacking *Hdac2*, indicating that HDAC1 and HDAC2 exhibit at least some distinct biological functions. In the current study, we found that cytotrophoblasts expressed HDAC1 and HDAC2, and that knockdown of either *HDAC1* or *HDAC2* did not impact syncytiotrophoblast formation. Interestingly, in cells with reduced *HDAC1* or *HDAC2*, we observed increased expression of the paralogous protein, which is consistent with results in other studies^40,49,50^. However, inhibition of both HDAC1 and HDAC2 using FK228, or knockdown of both *HDAC1* and *HDAC2*, inhibited cytotrophoblast differentiation, suggesting that HDAC1 and HDAC2 can compensate for each other during syncytiotrophoblast development.

In this study, we used three human cytotrophoblast models to assess acetylation patterns during the progression of syncytialization: primary cytotrophoblast cells isolated from term placentas, human trophoblast stem cells derived originally from first trimester placentas^25^, and BeWo trophoblasts. All three models of differentiating trophoblasts showed reduced histone acetylation at multiple lysine residues during the progression of syncytialization. Of note, experiments investigating the role of HDAC inhibition on syncytiotrophoblast development were performed primarily using BeWo trophoblasts, since isolated cytotrophoblasts harbor very few cycling cells and spontaneously initiate differentiation shortly following isolation, and culture conditions to maintain human trophoblast stem cells in an undifferentiated state require the use of the HDAC inhibitor, valproic acid. Although BeWo trophoblasts are a transformed line with certain limitations, they do possess the capacity to rapidly transition from a proliferative state to a differentiated state on stimulus, and exhibit gene expression changes, fusion, and hormone production reminiscent of the switch between cytotrophoblasts and fused syncytiotrophoblast in vivo. These cells are thus an advantageous tool for investigating molecular mechanisms associated with the conversion between cytotrophoblast proliferation and differentiation. Collectively, our findings indicate that histone deacetylation is likely a transient necessity for syncytialization, and is consistent with our in situ evidence that histone acetylation is apparent in a subset of syncytiotrophoblast nuclei.

In conclusion, we identified dynamic global and site-specific changes in AcH3 levels at key chromosomal regions during cytotrophoblast differentiation, which reveals new insights into regulatory mechanisms of gene expression changes during syncytiotrophoblast formation. It will be interesting to investigate these chromosomal regions, and genes located near these sites, for putative roles in syncytiotrophoblast development and function. It will also be interesting to uncover the role of histone deacetylation and HDAC1/HDAC2 activity in the regulation of extravillous trophoblast lineage development, and investigate the possibility of proteins other than histones whose activity is impacted by (de)acetylation. Our results caution against the use of HDAC inhibitors as front-line therapies in pregnant women diagnosed with cancer due to possible effects on placental function, and highlight the possibility of using clinically approved HDAC inhibitors in specific obstetric pathologies characterized by aberrant cytotrophoblast differentiation.

## Materials and methods

### Collection of human placentae

Placental tissues were obtained with written informed consent from patients undergoing elective termination of pregnancy (first trimester placenta samples) or elective cesarean section (term placenta samples) at London Health Sciences Centre (London, ON, Canada) following approval by the Research Ethics Boards at the University of Western Ontario and London Health Sciences Centre. Paraffin-embedded sections and flash-frozen samples of gestational age 6-week and 39-week human placenta were obtained from the Research Centre for Women’s and Infant’s Health Biobank (RCWIH, Mount Sinai Hospital, Toronto, ON, Canada, http://biobank.lunenfeld.ca), following appropriate consent, under protocols approved by Mount Sinai Hospital and the University of Western Ontario research ethics boards.

### Isolation of primary cytotrophoblast cells

Isolation of primary cytotrophoblast cells from term placenta was conducted according to the protocol by Kliman et al.^51^, with modifications. Briefly, villous tissue was thoroughly washed with phosphate buffered saline (PBS), scraped using a glass slide, and minced. Minced villous tissue was then digested in digestion buffer (Hank’s Balanced Salt Solution supplemented with 1 M HEPES, 7.5% sodium bicarbonate, 2.5% trypsin, and 150 kU deoxyribonuclease I, Sigma-Aldrich, Oakville, ON, Canada) at 37 °C for 30 min on a magnetic stirrer. Suspended cells were collected and immersed in fetal bovine serum (FBS, ThermoFisher Scientific, Mississauga, ON, Canada) to inhibit enzymatic activity. The remaining tissue was subjected to two additional 30 min cycles of enzymatic digestion. Cell suspensions were filtered through a 100 μm strainer to remove contaminating tissue debris, and then centrifuged at 1000 × *g* for 10 min. Cell pellets were resuspended in DMEM-F12 medium supplemented with 10% FBS, layered on top of a preformed Percoll gradient (consisting of dilutions of Percoll from 70 to 5%) and centrifuged at 1200 × *g* for 20 min without braking. The layer between 45 and 35% Percoll (containing cytotrophoblast cells) was collected, suspended in DMEM-F12 medium and centrifuged at 350 × *g* for 10 min. To remove residual non-cytotrophoblast cells, 10^7^ cells were resuspended in 100 μl MACS separation buffer, and incubated with 10 μl of phycoerythrin-conjugated anti-human leukocyte antigen-ABC antibody (catalog 130-120-431, Miltenyi Biotec, Auburn, CA) for 10 min at 4 °C. After washing, cells were resuspended in 80 μl buffer and incubated with 20 μl of anti-phycoerythrin-coated microbeads for 15 min at 4 °C. Cell pellets were washed and resuspended in 500 μl buffer. Cells expressing human leukocyte antigen-ABC (non-cytotrophoblast cells) were positively selected using a magnetic separation column and a MiniMACS^TM^ separator (Miltenyi Biotec). The remaining cells that passed through the column were counted, and cell viability was determined by trypan blue exclusion. We consistently achieved >90% viable cytotrophoblasts using this method. 2 × 10^5^ cytotrophoblasts/cm^2^ were cultured at 37 °C, 5% CO_2_ in DMEM-F12 media supplemented with 10% FBS, 100 units/ml penicillin, and 100 μM streptomycin (Sigma-Aldrich) for up to 72 h, during which time cells spontaneously formed syncytiotrophoblast.

### Culture of cell lines

Human trophoblast stem cells, derived from human 6-week placenta^25^, were maintained in DMEM-F12 media. Cells were passaged by using TrypLE (ThermoFisher Scientific) prior to reaching confluency and were maintained at 37 °C in an atmosphere consisting of 5% CO_2_ for no more than twenty sequential passages. Human trophoblast stem cells were maintained in a stem state, or induced to differentiate into syncytiotrophoblast for up to 5 days, using culture conditions described previously^25^.

BeWo trophoblasts are a well-characterized transformed human trophoblast cell line that exhibit cytotrophoblast characteristics^52^. BeWo cells were obtained from American Type Culture Collection (CCL-98, Manassas, VA), and were routinely tested by short tandem repeat profiling and for mycoplasma to authenticate cells and ensure consistency across passages. Cells were maintained at 37 °C, 5% CO_2_ in DMEM-F12 media supplemented with 10% FBS, 100 units/ml penicillin, and 100 μM streptomycin (Sigma-Aldrich). Differentiation of BeWo trophoblasts into syncytiotrophoblast was induced by exposing cells to a brominated, cell permeable derivative of cAMP, 8-Br-cAMP (Sigma-Aldrich, 250 μM) for up to 48 h, as we have done previously^53^.

Human embryonic kidney (HEK)-293T cells were obtained from American Type Culture Collection (CRL-3216). HEK-293T cells were maintained in DMEM supplemented with 10% FBS, 100 units/ml penicillin, and 100 μM streptomycin. Cells were passaged by light trypsinization prior to reaching confluency and were maintained at 37 °C with 5% CO_2_.

### Immunohistochemistry

Serial sections of human placental tissue were deparaffinized in Histoclear, and rehydrated using increasing dilutions of ethanol washes. Formaldehyde crosslinks were fragmented by placing slides in Reveal Decloaker (Biocare Medical, Pacheco, CA) at 95 °C for 20 min. Sections were then permeabilized using PBS containing 1% bovine serum albumin and 0.3% Triton-X, and nonspecific antibody binding was reduced by immersing slides in 10% normal goat serum (ThermoFisher Scientific). Sections were immersed in primary antibodies specific for AcH2BK5 (1:100, catalog 2574), AcH3K9 (1:800, catalog 9649), AcH3K27 (1:100, catalog 8173), AcH3K14 (1:400, catalog 7627), AcH3K18 (1:100, catalog 13998), CGB (1:100, catalog PA5-58598, ThermoFisher Scientific), and TEAD4 (1:50, catalog HPA056896, Sigma-Aldrich) overnight at 4 °C. Unless indicated otherwise, all antibodies were obtained from Cell Signaling Technology (Danvers, MA). The next day, sections were incubated for 1 h with species-appropriate fluorescent-conjugated antibodies (Alexa Fluor, ThermoFisher Scientific), and then incubated with 4′6-diamidino-2-phenylindole (DAPI, ThermoFisher Scientific) to detect nuclei. Slides were mounted using Fluoromount G (SouthernBiotech, Birmingham, AL) and imaged using a Nikon ECLIPSE Ni series microscope equipped with a Ds-Qi2 camera.

### Western blotting

Evaluation of protein expression was determined by western blotting. Cell lysates were prepared using radioimmunoprecipitation assay buffer (RIPA: 50 mM Tris, 150 mM NaCl, 1% NP-40, 0.5% sodium deoxycholate, 0.1% sodium dodecyl sulfate (SDS)). Approximately 50 μg of cell lysate was mixed with 4× reducing loading buffer (0.25 M Tris, 8% SDS, 30% glycerol, 0.02% bromophenol blue, 0.3 M dithiothreitol), boiled for 5 min, and subjected to SDS-polyacrylamide gel electrophoresis. Proteins were transferred to polyvinylidene difluoride membranes, blocked in tris buffered saline containing 3% bovine serum albumin and 0.5% Tween-20, and probed overnight at 4 °C using antibodies for AcH2BK5 (1:1000), AcH3K9 (1:1000), AcH3K27 (1:1000), AcH3K14 (1:1000), AcH3K18 (1:1000), acetylated H3 (1:10000, catalog 06-599, Sigma-Aldrich), total H3 (1:1000, catalog 499, Cell Signaling Technology), HDAC1 (1:1000, catalog 5356, Cell Signaling Technology), HDAC2 (1:1000, catalog 5113, Cell Signaling Technology), CGB (1:2000), E-cadherin (1:1000, catalog 14472, Cell Signaling Technology), and β-actin (1:4000, catalog sc47778, Santa Cruz Biotechnology, Santa Cruz, CA). Except where indicated, the antibodies used for western blotting are the same as those used for immunohistochemistry. All primary antibodies were diluted in tris buffered saline containing 0.5% Tween-20 and 3% bovine serum albumin. Membranes were then washed, incubated for 1 h with species-appropriate infrared-conjugated secondary antibodies (Cell Signaling Technology), and signals detected using a LI-COR Odyssey imaging system (LI-COR Biosciences, Lincoln, NE). Densitometric analysis of signal intensity was conducted using ImageJ (version 1.52a)^54^.

### Immunofluorescence

Cells were fixed with 4% paraformaldehyde and then permeabilized using PBS containing 0.3% Triton-X and 1% bovine serum albumin. Nonspecific antibody binding was blocked by immersing cells in 10% normal goat serum (ThermoFisher Scientific), and then cells were probed with mouse primary antibody specific for human E-cadherin (1:50, catalog 14472, Cell Signaling Technology) or rabbit primary antibody specific for cytokeratin 7 (1:100, catalog PA5-86169, ThermoFisher Scientific) overnight at 4°C. The next day, cells were incubated with Alexa Fluor 555 anti-mouse or anti-rabbit secondary antibodies for cells incubated with E-cadherin and cytokeratin 7, respectively. Cells that were previously immersed in the antibody targeting E-cadherin were then incubated with rabbit anti-human CGB (1:1000), followed by Alexa Fluor 488-conjugated anti-rabbit secondary antibody. Nuclei were then counterstained using DAPI. Images were captured using a Zeiss Axio fluorescence microscope. For quantification of trophoblast fusion, images were taken from three randomly selected fields per well in triplicate at ×20 magnification. The percent fusion was calculated by counting the total number of nuclei contained within fused cells (E-cadherin negative, CGB-positive) divided by the total number of nuclei. Cell counting was conducted using an in-house cell counting program designed in MATLAB.

### RT-PCR

RNA was extracted using Ribozol (VWR International, Mississauga, ON, Canada), according to the manufacturer’s instructions. RNA was converted into cDNA using reverse transcription (High Capacity cDNA kit, ThermoFisher Scientific), which was then diluted 1:10. Conventional PCR was conducted using primers described in Table 2, and DreamTaq DNA Polymerase (ThermoFisher Scientific) to amplify cDNA. Cycling conditions involved an initial holding step (95 °C for 3 min), followed by 33 cycles of PCR (95 °C for 30 s, 55–63 °C for 30 s, and 72 °C for 30 s), and a final elongation phase at 72 °C for 12 min. PCR products were resolved on 2% agarose gels and imaged using a ChemiDoc imaging system (Bio-Rad Laboratories, Mississauga, ON, Canada). Quantitative PCR was performed by amplifying cDNA using Sensifast SYBR Green PCR Master Mix (FroggaBio, Toronto, ON, Canada) and primers shown in Table 2. A CFX96 Connect real-time PCR detection system (Bio-Rad Laboratories) was used to detect fluorescence. Cycling conditions involved an initial holding step (95 °C for 10 min). Following this step, 40 cycles of two step PCR (95 °C for 15 s and 60 °C for 1 min) and a dissociation phase were performed. Relative mRNA expression was calculated using the ΔΔCt method^55^. The geometric mean from three constitutively expressed reference genes (*RNA18SN1, EEF2, YWHAZ*) was used as reference RNA.

**Table 2:** List of primers used for RT-PCR and chromatin immunoprecipitation (ChIP).

Gene	Forward	Reverse	Accession No.
*CGB*	CCTGGCCTTGTCTACCTCTT	GGCTTTATACCTCGGGGTTG	NM_000737.3
*ChIP-HSD11B2*	GGGACTGGACACTCAACAGG	AGAACTCTCCCACTCTTGCG	NC_000016.10
*ChIP-OVOL1*	CCACCCTCACCTGTGTTTGA	GGCTCAGCTCACCTTTACCA	NC_000011.10
*EEF2*	AGGCGTAGAACCGACCTTTG	GACAGCGAGGACAAGGACAA	NM_001961.4
*ERVV1*	TAACAGTGGGGCGATAGAGG	AGACTTCACAGCCTCCCAAA	NM_152473.2
*ERVV2*	CAGGCACAGTGGAATGAAAA	GACCTGGTGATGAAGTTGTGG	NM_001191055.2
*ERVFRD-1*	CCAAATTCCCTCCTCTCCTC	CGGGTGTTAGTTTGCTTGGT	NM_207582.3
*ERVW1*	CTACCCCAACTGCGGTTAAA	GGTTCCTTTGGCAGTATCCA	NM_001130925.2
*HDAC1*	TCGATCTGCTCCTCTGACAA	GCTTCTGGCTTCTCCTCCTT	NM_004964.3
*HDAC10*	ATGGCCAGGGGATCCAGTAT	TTTGCCCCTCAAAGGCCAGT	NM_001159286.2
*HDAC2*	TGTGCCTCAGTTGCTTCATC	GATGCAGTGAGCCAAGATCA	NM_001527.4
*HDAC3*	GGAGCTGGACACCCTATGAA	GACTCTTGGTGAAGCCTTGC	NM_001355039.2
*HDAC4*	CAAGCACCCCTCGTCACAG	GCCTCTTCCTCATCGCTCTC	NM_006037.3
*HDAC5*	AGCAAAAGCCCAACATCAAC	AACTTCTGCACACAGCTCCA	NM_001015053.2
*HDAC6*	AGGTCGCCAGAAACTTGGTG	TGGGGGTTCTGCCTACTTCT	NM_001321225.2
*HDAC7*	CTCTCGCCGTCTCACAGTC	TCGCTTGCTCTTGTCCTTGT	NM_001098416.4
*HDAC8*	AAGCAGATGAGAGATGAAGCA	TGCCAATTCCCACTGGAGTC	NM_001166418.2
*HDAC9*	CCTTTTTGCTTCTGCCTCACC	CAGCCACAGAATAGCACCCA	NM_001204144.3
*HSD11B2*	CAGATGGACCTGACCAAACC	AGCTCCGCATCAGCAACTAC	NM_000196.4
*OVOL1*	CCGTGCGTCTCCACGTGCAA	GGCTGTGGTGGGCAGAAGCC	NM_004561.4
*RNA18SN1*	GCAATTATTCCCCATGAACG	GGCCTCACTAAACCATCCAA	NR_145820.1
*TEAD4*	CAGTATGAGAGCCCCGAGAA	TGCTTGAGCTTGTGGATGAA	NM_003213.4
*TP63*	CAGATGGACCTGACCAAACC	AGCTCCGCATCAGCAACTAC	NM_001114978.2
*YWHAZ*	ATGCAACCAACACATCCTATC	GCATTATTAGCGTGCTGTCTT	NM_001135699.1

### Chromatin immunoprecipitation sequencing

Chromatin immunoprecipitation was performed as previously described^53^. Briefly, cells cultured under control and differentiated conditions were fixed with 0.7% formaldehyde for 10 min, and purified nuclear lysates were sonicated using a Bioruptor (Diagenode, Denville, NJ) to prepare DNA fragments at a size of ~200–400 bp. Approximately 1% of sonicated nuclear lysate was removed to serve as an input control. Half of the remaining lysate was incubated with AcH3 antibody (5 μg, Sigma-Aldrich catalog 06-599) overnight at 4 °C. The other half was incubated with rabbit IgG (5 μg, catalog 2729, Cell Signaling Technology) overnight at 4°C to serve as a nonspecific binding control. The next day, immunoprecipitated chromatin fragments were captured using protein G-conjugated Sepharose beads (Sigma-Aldrich). Eluted and purified DNA fragments were amplified using DreamTaq DNA polymerase (ThermoFisher Scientific) and primers described in Table 2, or analyzed using an Agilent Bioanalyzer (Agilent Technologies, Santa Clara, CA), quantified using Qubit, and libraries generated using a NEBNext Ultra II DNA Library Prep Kit (New England Biolabs, Whitby, ON, Canada). Libraries were sequenced on an Illumina (San Diego, CA) NextSeq High Output at the London Regional Genomics Facility using 75-bp single end reads, which generated at least 42 million reads per sample.

Raw reads were trimmed with the TrimGalore wrapper script around the sequence-grooming tool cutadapt version 0.4.1 with the following quality trimming and filtering parameters^56^. The trimmed reads were mapped onto the hg19 reference genome downloaded from UCSC genome browser^57^, using HISAT2 version 2.0.4 (‘–no-spliced-alignment’)^58^. SAMtools (http://samtools.sourceforge.net/) was then used to convert SAM files and index BAM files. BigWig coverage tracks were generated using deepTools2 from the aligned reads^59^. The coverage was calculated as the number of reads per 50 bp bin and normalized by 1× sequencing depth (effective hg19 genome size = 2,509,729,011).

A sliding window approach implemented in the csaw R/Bioconductor package was used to identify regions enriched in AcH3^60^. In brief, reads outside of blacklist regions (https://github.com/Boyle-Lab/Blacklist) with mapq ≥ 20 were counted inside 200-bp sliding windows. Global background was estimated by counting reads within contiguous 2000-bp bins across the genome. Windows were filtered out if the fold enrichment was below log_2_(3) compared with background. Filtered windows less than 100 bp apart were merged and a maximum region size of 5000 bp was permitted, conferring a total of 49,648 regions. We performed differential binding analysis of count data within these regions using DESeq2 R/Bioconductor package^61^, and used the independent hypothesis weighting R/Bioconductor package to weight *P*-values and adjust for multiple testing^62,63^. Raw and processed data is deposited in NCBI’s Gene Expression Omnibus (GSE141867)^64^.

### Treatment of trophoblasts with HDAC small molecule inhibitors

BeWo trophoblasts were pretreated with pan and specific HDAC inhibitors. After 2 h of HDAC inhibitor treatment, the cells were induced to differentiate by treating with 250 μM 8-Br-cAMP. The doses and specificity of the inhibitors used in this study are detailed in Table 1. All inhibitors were dissolved initially in dimethyl sulfoxide (DMSO) except TSA, which was dissolved initially in ethanol. Thus, additional controls were included in which cells were exposed to either 0.1% DMSO or 0.005% ethanol (the highest concentrations of vehicle in which cells were exposed during experiments). For all inhibitors, there was no obvious effect on cell viability at the doses used, nor did the vehicle in which the inhibitors were dissolved affect cytotrophoblast viability or differentiation.

### Lentivirus production and shRNA-mediated gene knockdown

Two-distinct HDAC1 and HDAC2 knockdown shRNA constructs encoded in PLKO.1 vectors were obtained from Sigma-Aldrich (HDAC1-KD1*: CCTAATGAGCTTCCATACAAT*, HDAC1-KD2*: CGGTTAGGTTGCTTCAATCTA*, HDAC2-KD1*: CAGTCTCACCAATTTCAGAAA*, HDAC2-KD2*: CAGACTGATATGGCTGTTAAT*). Control shRNA (scrambled) constructs that do not target any known mammalian genes were obtained from Addgene (plasmid 1864, Cambridge, MA). To establish HDAC1/HDAC2 DKD, the puromycin resistance cassettes encoded within the shHDAC1-KD1 and Control PLKO.1 vectors were replaced with a cassette encoding blasticidin resistance. Lentiviral plasmids (MD2.G, MDLG/RRE, and RSV-Rev) were used to produce lentivirus, as previously described^65^. HEK293T cells were transfected using Lipofectamine 2000 (ThermoFisher Scientific) in Opti-MEM medium (ThermoFisher Scientific) with the shRNA containing vectors and packaging plasmids. Culture supernatants containing lentivirus were collected every 24 h for a total of 48 h, centrifuged, and stored at −80 °C until use.

Human BeWo trophoblasts were exposed to lentiviral particles for 48 h in the presence of hexadimethrine bromide (8 μg/ml, Sigma-Aldrich) diluted in normal growth medium. After 48 h, transduced cells were selected using puromycin (3.5 μg/ml, ThermoFisher Scientific) or blasticidin (2.5 μg/ml, ThermoFisher Scientific) for at least 48 h. A negative control well containing cells that were not exposed to virus was used to ensure efficient transduction.

### Enzyme immunoassay

The concentration of CGB in supernatants of primary cytotrophoblasts was measured by an enzyme immunoassay (catalog DY9034-05, R&D Systems, Minneapolis, MN), using a protocol provided by the manufacturer. Briefly, conditioned media were collected from trophoblast cultures, centrifuged to remove debris, and supernatants added to a microplate precoated with a monoclonal antibody specific to CGB. After washing, a biotinylated rabbit anti-human CGB antibody was added, followed by streptavidin conjugated to horseradish peroxidase. Excess solution was washed, and the peroxidase substrate 3,3′,5,5′-tetramethylbenzidine was added to the microplate to generate a colorimetric reaction. The reaction was stopped by adding 2 N H_2_SO_4_, and absorbance was measured in a spectrophotometer at 450 nm. To calculate the concentration of CGB in trophoblast supernatants, absorbance was compared with a standard curve, generated by graphing absorbance values relative to known CGB concentrations.

### Statistical analysis

For isolation of primary cytotrophoblasts, four different placentas were used. All placentas were deemed to be from uncomplicated pregnancies, and there were no major differences in the number or quality of cytotrophoblasts from each placenta. For chromatin immunoprecipitation-sequencing experiments, immunoprecipitated DNA from three independent replicates of undifferentiated cells and three independent replicates of differentiated cells were used. Inputs from undifferentiated and differentiated cells from each experiment were combined prior to sequencing. All other experiments were repeated at least three independent times. Variance was similar between groups being statistically compared. Statistical comparisons for densitometry analyses were tested using one sample *t*-test, all other comparisons were tested using analysis of variance, followed by Tukey’s post-hoc test. Means were considered statistically different if *P* < 0.05. GraphPad Prism 6.0 and MATLAB were used for all graphing and statistical analyses. All graphs represent means ± SEM.

## Supplementary information

Supplementary Figure Legends

Fig S1

Fig S2

Fig S3

Fig S4

Fig S5
