# Supplementary material for: Histone deacetylase 1 and 2 drive differentiation and fusion of progenitor cells in human placental trophoblasts
Source: Cell Death Dis. 2020 May 4;11(5):311. doi: 10.1038/s41419-020-2500-6 (PMC7198514; doi:10.1038/s41419-020-2500-6)
Supplement: Supplementary file 1 — Supplementary Figure Legends [file 41419_2020_2500_MOESM1_ESM.docx]

**Supplementary Figure Legends**

**Fig S1. Genomic regions harboring dynamic changes in AcH3 binding proximate to genes associated with syncytiotrophoblast development.** Representative genome browser views of chromosomal regions proximate to *ERVW1*, *ERVFRD-1*, and *HSD11B2,* which exhibit differential AcH3 binding in BeWo trophoblasts exposed to undifferentiated and differentiated conditions.

**Fig S2. Effect of the broad-spectrum HDAC inhibitor SAHA on histone acetylation and syncytiotrophoblast development.** BeWo trophoblasts were exposed to SAHA (50-750 nM), and then induced to differentiate for 48 h. A) Levels of AcH2BK5, AcH3K27, AcH3K14, and total histone H3 were determined by western blotting. B) Transcript levels of *ERVW1, ERVFRD-1*, *CGB,* and *HSD11B2* in cells cultured in undifferentiated (black bar) and differentiation (blue bars) conditions with or without various doses of SAHA. C) Percentage of fused cells following culture in undifferentiated or differentiation conditions with or without 750 nM SAHA. Representative images of E-cadherin (red) and CGB (green) are shown to the left of the graph. Nuclei were counterstained using DAPI (blue). Graphs represent means ± SEM. Data significantly different from Ctrl (0 nM SAHA) cells cultured in differentiation conditions are indicated by an asterisk (*; P<0.05; n=4 in panel B, n=9 images from 3 experiments in panel C). Scale bars represent 80 μm.

**Fig S3. Effect of reduced HDAC1 expression on trophoblast differentiation.** A) Expression of *HDAC1* and *HDAC2* in BeWo trophoblasts expressing control (scrambled, SCR) shRNA or two distinct shRNAs targeting HDAC1 (KD1 and KD2), cultured in undifferentiated (black bars) and differentiation (blue bars) conditions. B) Western blot showing protein levels of HDAC1 and HDAC2 in SCR, KD1 and KD2 cells cultured in undifferentiated and differentiation conditions. β-Actin was used as a loading control. C) Transcript levels of *ERVFRD-1*, *HSD11B2*, *CGB* and *TP63* in SCR, KD1, and KD2 cells cultured in undifferentiated and differentiation conditions. D) Percentage of SCR, KD1, and KD2 cells that underwent syncytialization (E-cadherin-negative, CGB-positive) following culture in undifferentiated or differentiation conditions. Representative images of E-cadherin (red) and CGB (green) are shown above the graph. Nuclei were counterstained using DAPI (blue). Scale bars represent 80 μm. Graphs represent means ± SEM. Data significantly different from SCR are indicated by an asterisk (*, P<0.05, n=at least 3 in panels A and C, n=9 images from 3 experiments in panel D). Statistical comparisons are shown only for differentiation conditions.

**Fig S4. Effect of reduced HDAC2 expression on trophoblast differentiation.** A) Expression of *HDAC1* and *HDAC2* in BeWo trophoblasts expressing control (scrambled, SCR) shRNA or two distinct shRNAs targeting HDAC2 (KD1 and KD2), cultured in undifferentiated (black bars) and differentiation (blue bars) conditions. B) Western blot showing protein levels of HDAC1 and HDAC2 in SCR, KD1 and KD2 cells cultured in undifferentiated and differentiation conditions. β-Actin was used as a loading control. C) Transcript levels *of ERVFRD-1, HSD11B2, CGB,* and *TP63* in SCR, KD1, and KD2 cells cultured in undifferentiated and differentiation conditions. D) Percentage of SCR, KD1, and KD2 cells that underwent syncytialization (E-cadherin-negative, CGB-positive) following culture in undifferentiated or differentiation conditions. Representative images of E-cadherin (red) and CGB (green) are shown above the graph. Nuclei were counterstained using DAPI (blue). Scale bars represent 80 μm. Graphs represent means ± SEM. Data significantly different from SCR are indicated by an asterisk (*, P<0.05, n=at least 3 in panels A and C, n=9 images from 3 experiments in panel D). Statistical comparisons are shown only for differentiation conditions.

**Fig S5. Decreased AcH3 at the transcription start site of *OVOL1* and *HSD11B2* in differentiating trophoblasts following knockdown of HDAC1 and HDAC2.** Chromatin immunoprecipitation was performed using fixed, sonicated lysates of BeWo trophoblasts expressing control (scrambled, SCR) shRNA or shRNAs targeting HDAC1 and HDAC2 (double knockdown, DKD), cultured in differentiation conditions. AcH3 was immunoprecipitated, DNA purified, and PCR conducted to determine AcH3 enrichment near the transcription start site (TSS) of *OVOL1* and *HSD11B2*. 1% chromatin (input) was used as a positive control; immunoprecipitation with non-specific rabbit IgG was used as a negative control. A schematic showing where the primers (arrowheads) were designed in relation to the TSS is shown on the left.
