# Supplementary figures and images for: Histone deacetylase 1 and 2 drive differentiation and fusion of progenitor cells in human placental trophoblasts
Source: Cell Death Dis. 2020 May 4;11(5):311. doi: 10.1038/s41419-020-2500-6 (PMC7198514; doi:10.1038/s41419-020-2500-6)

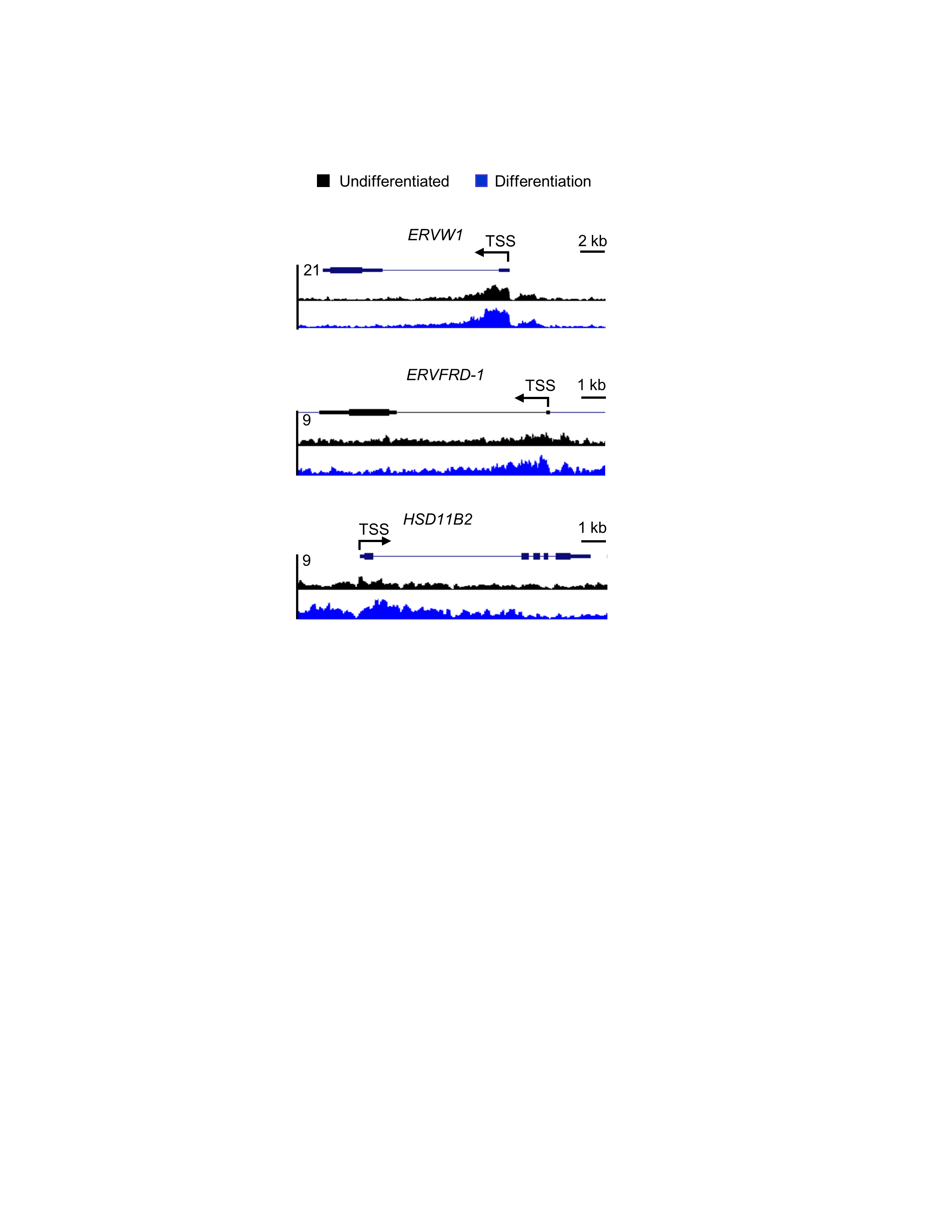

Supplement: Supplementary file 2 — Fig S1 [file 41419_2020_2500_MOESM2_ESM.tif]

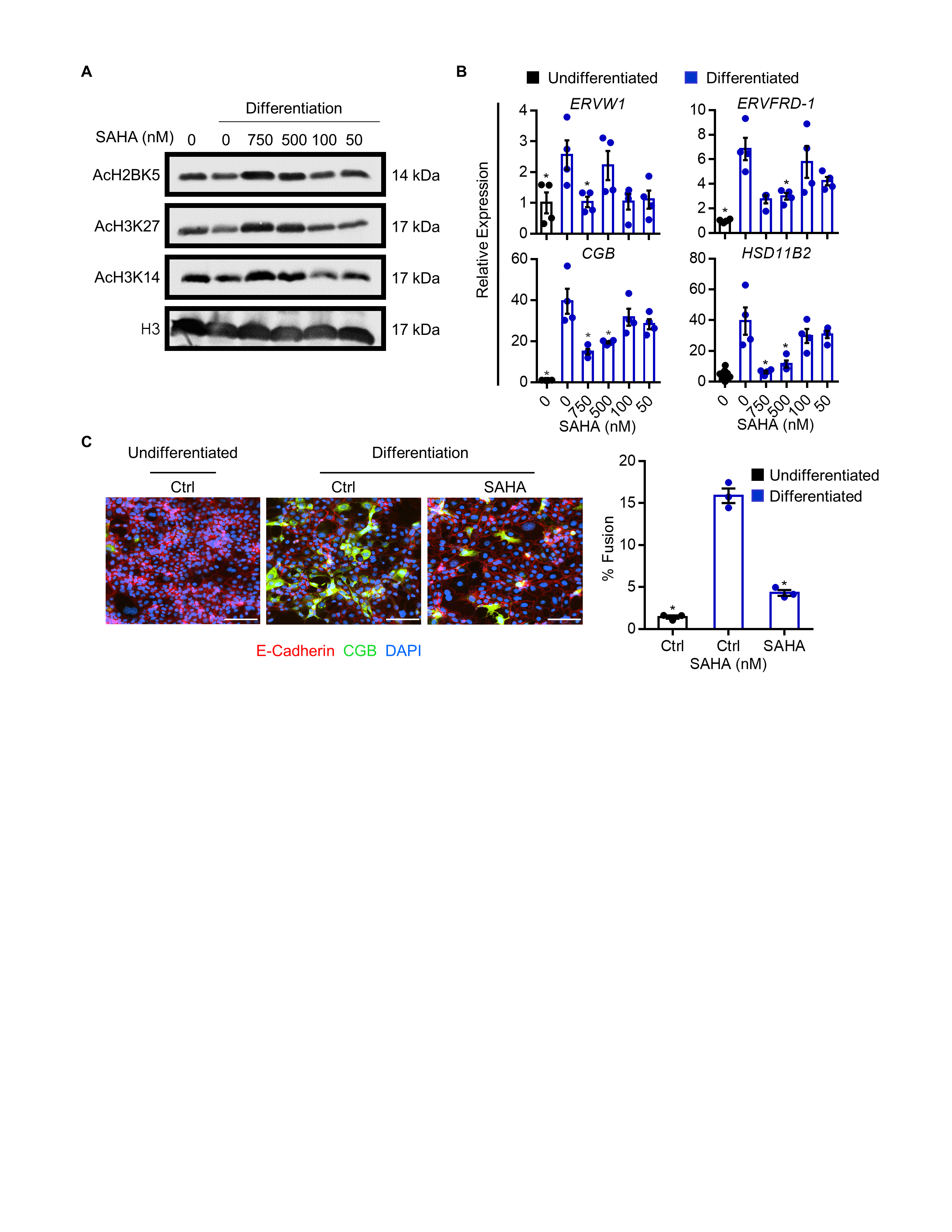

Supplement: Supplementary file 3 — Fig S2 [file 41419_2020_2500_MOESM3_ESM.tif]

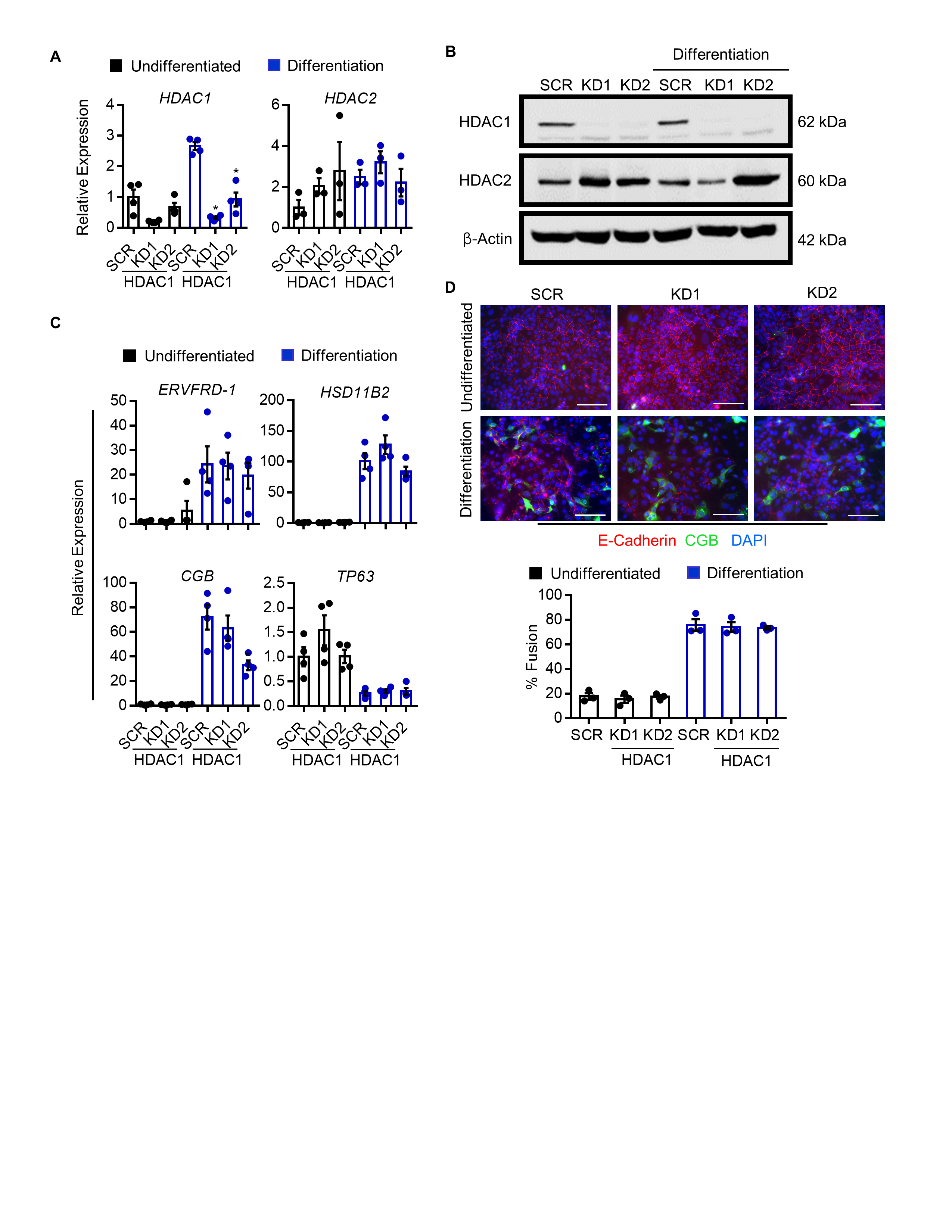

Supplement: Supplementary file 4 — Fig S3 [file 41419_2020_2500_MOESM4_ESM.tif]

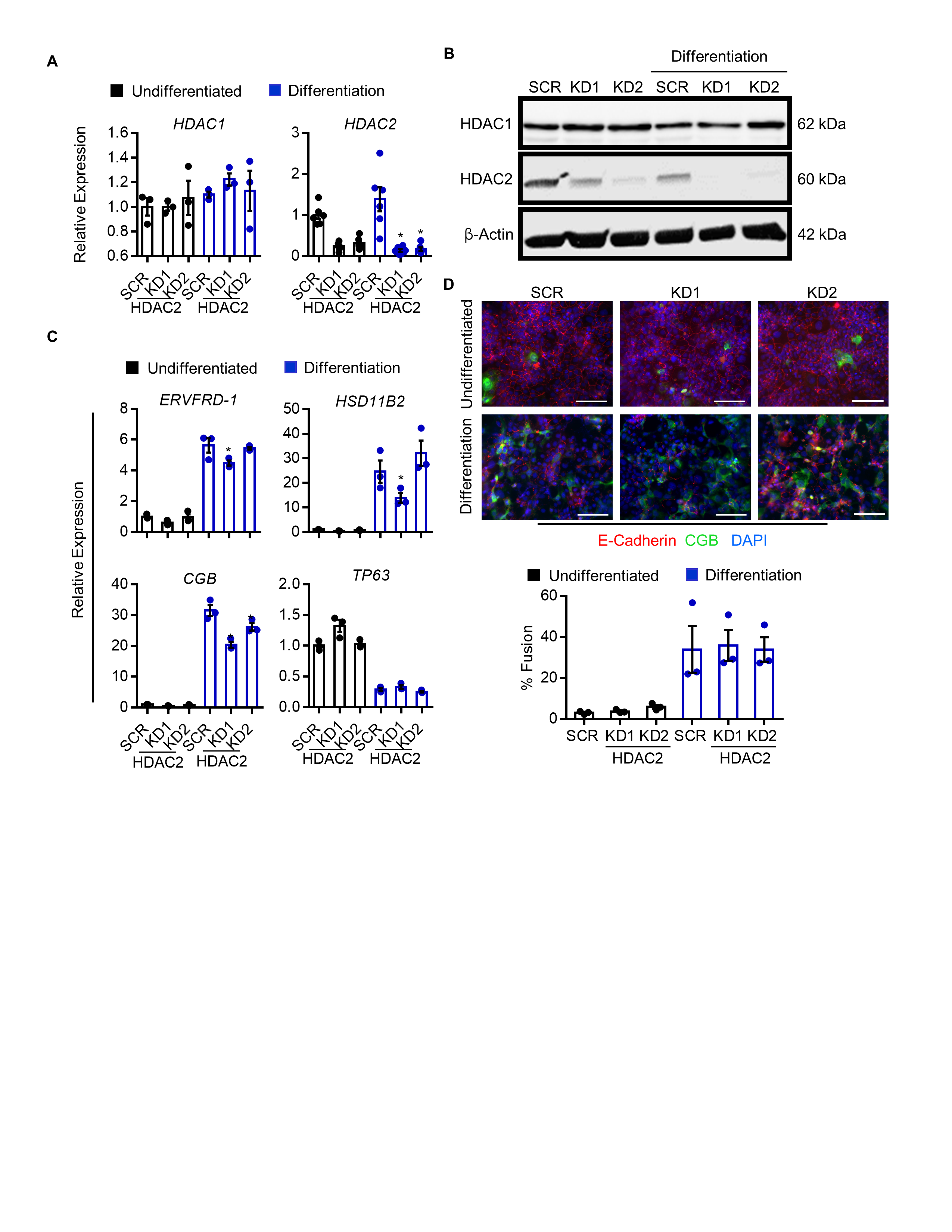

Supplement: Supplementary file 5 — Fig S4 [file 41419_2020_2500_MOESM5_ESM.tif]

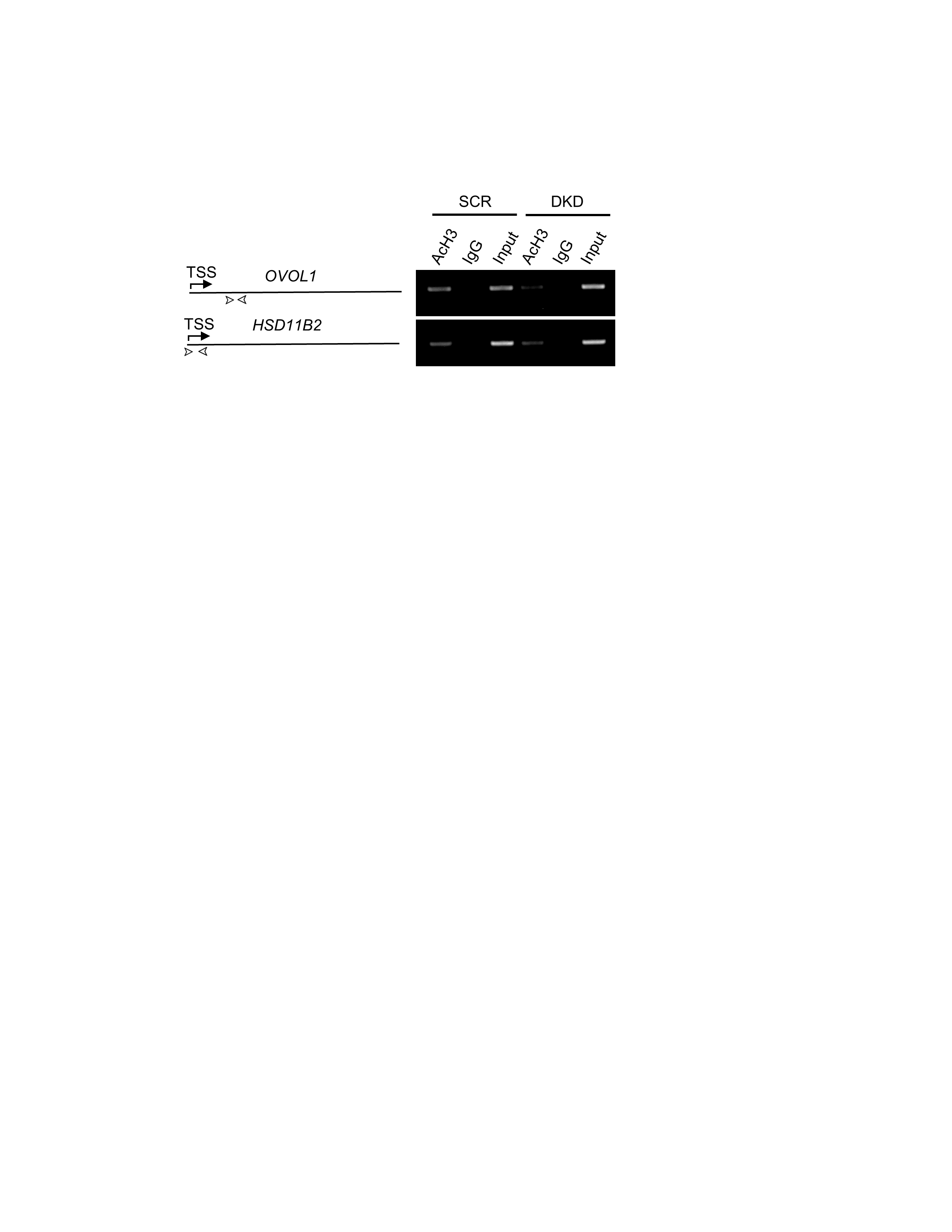

Supplement: Supplementary file 6 — Fig S5 [file 41419_2020_2500_MOESM6_ESM.tif]
